# Causal relationship between diet and knee osteoarthritis: A Mendelian randomization analysis

**DOI:** 10.1371/journal.pone.0297269

**Published:** 2024-01-31

**Authors:** Xiaofeng Lv, Fangqi Liang, Shanshan Liu, Xinmin Deng, Rui Lai, Jihang Du, Jian Luo

**Affiliations:** 1 School of Acupuncture and Tuina, Chengdu University of Traditional Chinese Medicine, Chengdu, China; 2 Hospital of Chengdu University of Traditional Chinese Medicine, Chengdu, Sichuan, China; Majmaah University College of Applied Medical Sciences, SAUDI ARABIA

## Abstract

**Background:**

Knee osteoarthritis (KOA) is a common disabling joint disease that affects millions of people worldwide. Diet may play a role in the etiology and progression of KOA, but evidence for a causal relationship is limited. We aimed to investigate the causal impact of dietary intake on KOA risk using Mendelian randomization (MR).

**Methods:**

We used summary-level data from genome-wide association studies (GWAS) including dietary intake (n = 335, 394–462, 342), and KOA (n = 403, 124). We selected 6–77 genetic variants as instrumental variables for 18 dietary factors, including processed meat, poultry, beef, oily fish, non-oily fish, pork, lamb, frequency of alcohol intake, alcoholic beverages, tea, coffee, dried fruit, cereals, cheese, bread, cooked vegetables, salad/raw vegetables, and fresh fruit. We performed univariate and multivariate MR analyses to estimate the causal effect of each dietary factor on KOA risk. We also performed some sensitivity analyses to assess the validity of the MR hypothesis.

**Results:**

We found that higher coffee intake was associated with increased KOA risk, whereas higher intake of dried fruits, grains, cheese, and oily fish was associated with reduced KOA risk. After multivariate adjustment, we found that coffee and oily fish intake may affect KOA through obesity, body mass index (BMI), diabetes, hypertension, and prolonged standing. Sensitivity analyses did not reveal any evidence of pleiotropy.

**Conclusions:**

Our study provides new causal evidence that dietary intake may influence KOA risk. Specifically, we suggest that increased intake of dried fruits, grains, cheese, and oily fish and decreased coffee intake may be beneficial in preventing and mitigating KOA. further studies are needed to elucidate the underlying mechanisms and to confirm our findings in different populations.

## 1 Introduction

Knee osteoarthritis (KOA) is a common degenerative joint disease that affects millions of people worldwide [1]. It often causes pain, stiffness, and reduced mobility of the affected joint, severely affecting the quality of life and social functioning of patients [2]. According to the 2019 Global Burden of Disease Study, KOA is the sixth leading cause of life years of disability worldwide and the fourth leading cause of life years of disability in China [3]. The prevalence and disease burden of KOA is on the rise with the aging population and increasing obesity rates [4]. The etiology of KOA is multifactorial, involving genetic, biomechanical, and environmental factors. Among the environmental factors, diet has been suggested to play a role in the development and progression of KOA, but the evidence is inconclusive and inconsistent [5].

There are studies that show eating a Mediterranean diet with more vegetables and fruits, whole grain cereals, and nuts can reduce the risk of KOA and decrease pain symptoms [6]. In addition, high-fat diets such as oils accelerate cartilage wear and increase the risk of KOA [7]. In a cross-sectional observational study in Korea, the prevalence of KOA was found to increase with increased coffee intake [8]. Diet may influence KOA through several mechanisms, such as modulating inflammation, oxidative stress, cartilage metabolism, bone health, and body weight [9–11]. However, different dietary components may have different effects on KOA, and the optimal dietary pattern for preventing or treating KOA is unclear. Moreover, most of the previous studies on diet and KOA are observational, which cannot establish causal relationships and are prone to confounding and bias.

Therefore, there is a need for rigorous and robust studies to examine the causal effects of diet on KOA. One of the most powerful methods to infer causality is the Mendelian randomization (MR) analysis, which uses genetic variants as instrumental variables to mimic randomized controlled trials [12]. MR analysis can overcome some of the limitations of observational studies, such as reverse causation and residual confounding, by exploiting the random allocation of genes at conception.

In this study, we aimed to investigate the causal effects of various dietary factors on KOA using a two-sample MR analysis. Since obesity [13], body mass index (BMI) [14], hypertension [15], diabetes [16] and prolonged standing [17] are known risk factors for KOA. We also used multivariate correction to make the results more robust. The potential mechanisms of dietary effects on KOA were explored to provide a theoretical basis for KOA prevention.

## 2 Materials and methods

**Fig 1:**
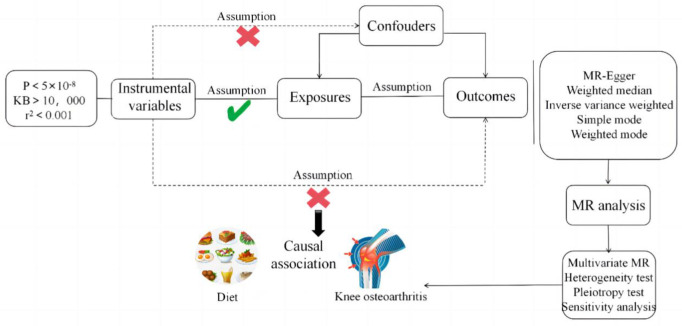
Flowchart of MR analysis.

To be a valid instrument for causal inference in MR studies, genetic variation should meet three key assumptions. First, genetic variation must be robustly associated with exposure (Diet). Second, genetic variation must be independent of exposure-outcome confounders. Third, genetic variation must affect outcome (KOA) only through exposure (Diet), with no other pleiotropic effects (Fig 1). We performed this MR study using a previously published, publicly accessible, large-scale GWAS summary dataset [18]. All participants gave written informed consent in the original GWAS.

### 2.1 Data source

We obtained diet-related exposure data from the IEU Open GWAS project (https://gwas.mrcieu.ac.uk), comprising vegetable intake, meat intake, beverage intake, fruit intake, and staple food intake, for a total of 18 exposures. The exposure data were of European origin, with sample sizes ranging from 335,394 to 462,342. We selected exposure data with large sample sizes and high sequencing depth, which have been used in other MR studies [19–21]. The IEU Open GWAS project extracted these GWAS summary data directly or indirectly from the UK Biobank (http://www.nealelab.is/uk-biobank).

We used KOA summary data from the UK Biobank study of European ancestry, including 24,955 cases and 378,169 controls. The study analyzed four phenotypes related to osteoarthritis and identified putative effector genes by integrating eQTL co-localization, sound localization, animal model, and osteoarthritis tissue expression data [18]. The study also detected novel loci associated with osteoarthritis. Table 1 shows details on the exposures and outcome data used in this study.

**Table 1:** Information on exposures and outcome data.

GWAS ID	Exposure or outcome	IVs	Sample size	Population
ieu-b-73	Alcoholic drinks per week	27	335394	European
ukb-b-5779	Alcohol intake frequency	77	462346	European
ukb-b-6324	Processed meat intake	19	461981	European
ukb-b-8006	Poultry intake	6	461900	European
ukb-b-2826	Beef intake	11	461053	European
ukb-b-17627	Non-oily fish intake	9	460880	European
ukb-b-2209	Oily fish intake	46	460443	European
ukb-b-5640	Pork intake	11	460162	European
ukb-b-14179	Lamb/mutton intake	25	460006	European
ukb-b-11348	Bread intake	25	452236	European
ukb-b-1489	Cheese intake	53	451486	European
ukb-b-8089	Cooked vegetable intake	11	448651	European
ukb-b-6066	Tea intake	38	447485	European
ukb-b-3381	Fresh fruit intake	43	446462	European
ukb-b-15926	Cereal intake	29	441460	European
ukb-b-1996	Salad/raw vegetable intake	14	435435	European
ukb-b-5237	Coffee intake	31	428860	European
ukb-b-16576	Dried fruit intake	30	421764	European
ebi-a-GCST007090	Knee osteoarthritis	NA	24955 cases and 378169 controls	European

IV, Instrumental variable. SNP, Single nucleotide polymorphism. GWAS, Gene-wide association group. NA, Not applicable.

### 2.2 Instrumental variable selection

We applied uniform screening criteria to the instrumental variables (IVs) we used. The genome-wide significance threshold was P < 5 × 10^−8^. To avoid bias due to linkage disequilibrium (LD), we only included SNPs significantly associated with the exposure that had r^2^ < 0.001 and KB > 10,000 in the final analysis. We manually removed SNPs associated with KOA using PhenoScanner (http://www.phenoscanner.medschl.cam.ac.uk) to eliminate the interference of known confounders on causality estimates. These confounders included obesity, BMI, hypertension, diabetes and prolonged standing. We also used the F-statistic to quantify the strength of the genetic instrument and excluded SNPs with an F-statistic below 10 [22].

### 2.3 Statistical analyses

Statistical analysis was performed using the R programming language (version 4.1.2). MR analysis was based on the "TwoSampleMR" package (version 0.5.6). And the "MRPRESSO" package (version 1.0) is used to apply MRPRESSO analysis to identify SNPs with abnormal values and to delete abnormal SNPs for re-running MR analysis.

To determine the causal relationship between different diets and KOA, we performed a two-sample MR method using the above data. We used inverse variance weighting (IVW) as the primary analysis method [23], and MR-Egger, weighted median, simple mode and weighted mode as secondary methods [24, 25]. We also tested for heterogeneity and pleiotropy of the IVs. We assessed heterogeneity using Cochran Q with IVW and MR-Egger regression [26]. Horizontal pleiotropy could bias our results, so we estimated it using the MR-Egger intercept method and checked whether the intercept term after linear regression analysis differed from zero [27]. We conducted leave-one-out analysis to examine the influence of individual IVs on the results. We applied MR-PRESSO analysis with 5000 permutations to detect and remove outliers [28]. We repeated the MR analysis after removing the outliers.

### 2.4 Multivariate Mendelian randomization analyses

To investigate the direct causal effect of diet on KOA, we performed Multivariate Mendelian randomization (MVMR) analyses. In previous studies, it was found that KOA may be strongly influenced by obesity [29], hypertension [15], diabetes mellitus [30], and prolonged standing [31]. Therefore, we corrected for the potential interference of obesity, BMI, hypertension, diabetes and prolonged standing in the MVMR analysis.

## 3 Results

### 3.1 Selection of instrumental variables

First, we extracted 18 publicly available diet related GWAS summary datasets using R language. To ensure that the selected IVs were strongly correlated with exposure, we set the following parameters: P < 5E-08, r^2^ < 0.001 and KB > 10,000. We then manually excluded SNPs affected by confounding factors and removed outlier SNPs using MR-PRESSO. Finally, we identified 6–77 SNPs as IVs, details of which are provided in S1 File. The F-statistics of the final analyzed IVs were all >10, indicating a low likelihood of weak instrumental variable bias.

### 3.2 Univariate and multivariate Mendelian randomization analysis

**Fig 2:**
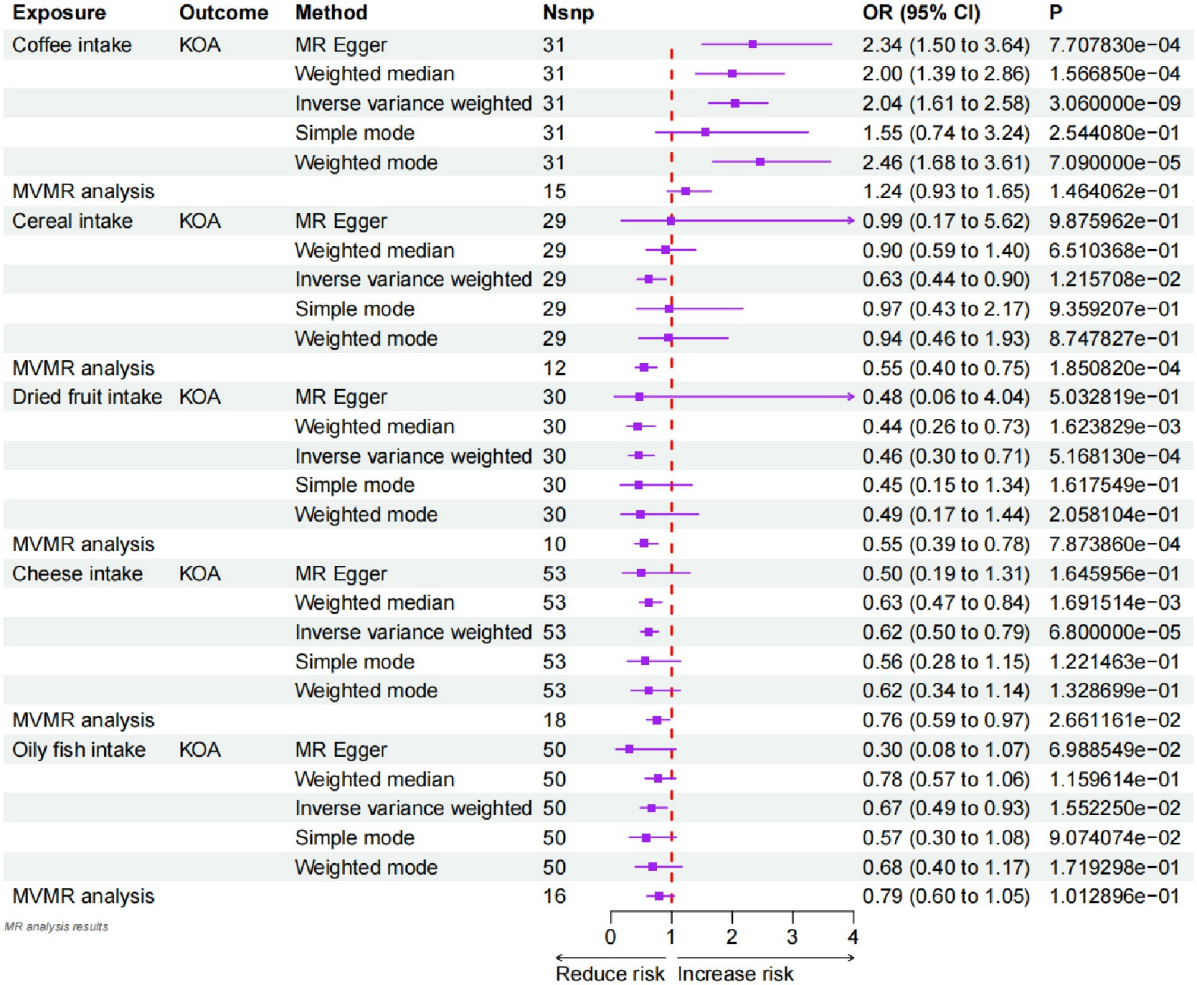
Forest plot of the causal relationship between diet and knee osteoarthritis. Positive results for the causal relationship between diet and KOA (coffee intake, cereal intake, dried fruit intake, cheese intake, oily fish intake). An OR value greater than 1 suggests that the exposure indicator is a risk factor, while the opposite is a protective factor. KOA: Knee osteoarthritis.

Using univariate Mendelian randomization (UVMR), we identified causal associations between five dietary factors and KOA. We applied the IVW method as the primary analysis and found that coffee consumption increased the risk of KOA (odds ratio [OR], 2.04; 95% confidence interval [CI], 1.61–2.58; P = 3.060E-09) (Fig 2). We also observed that cereal, dried fruit, cheese, and oily fish intake decreased the risk of KOA (Cereal intake: OR, 0.63; 95%CI, 0.44–0.90; P = 0.012. Dried fruit intake: OR, 0.46; 95%CI, 0.30–0.71; P = 0.0005. Cheese intake: OR, 0.62; 95%CI, 0.50–0.79; P = 6.800E-05. Oily fish intake: OR, 0.67; 95%CI, 0.49–0.93; P = 0.016.) (Fig 2). However, we did not detect any associations between KOA risk and meat consumption (processed meat, poultry, beef, non-oily fish, pork, and lamb), alcohol intake frequency, alcoholic drinks per week, tea consumption, bread intake, cooked vegetable intake, salad/raw vegetable intake, or fresh fruit intake (S1 File). To examine the direct causal effects of cereal, dried fruit, cheese, oily fish, and coffee intake on KOA, we performed multivariable Mendelian randomization (MVMR) analysis. After adjusting for obesity, BMI, hypertension, diabetes, and prolonged standing the results for oily fish and coffee intake became non-significant, while the other results remained consistent (Fig 2).

### 3.3 Sensitivity analysis

**Fig 3:**
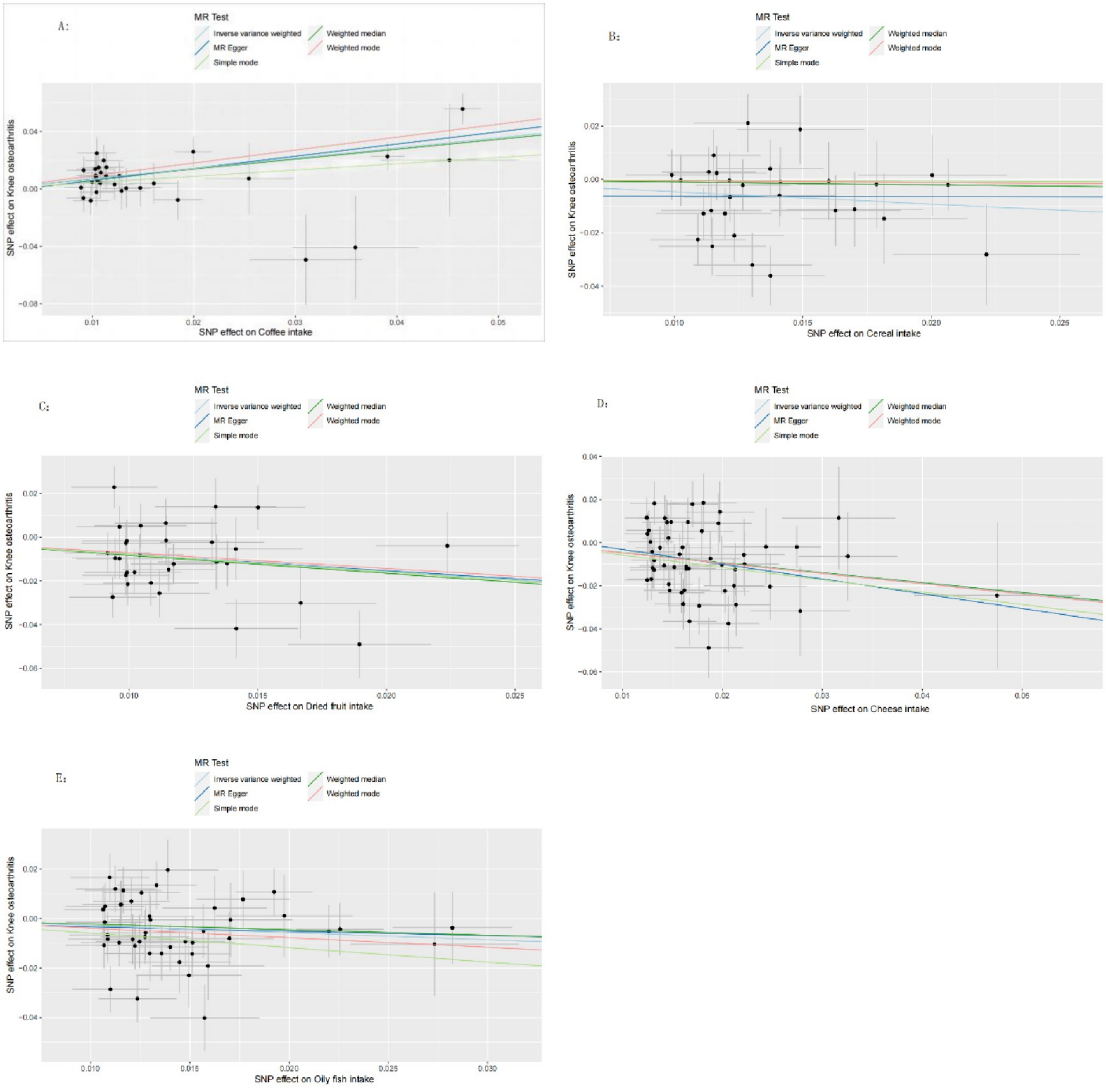
Scatter plot of MR analysis. Scatter plot of genetic correlation between diet and KOA by different MR analysis methods. Figures A, B, C, D, and E show the causal associations between coffee intake, cereal intake, dried fruit intake, cheese intake, and oily fish intake and KOA, respectively. KOA: Knee osteoarthritis.

**Fig 4:**
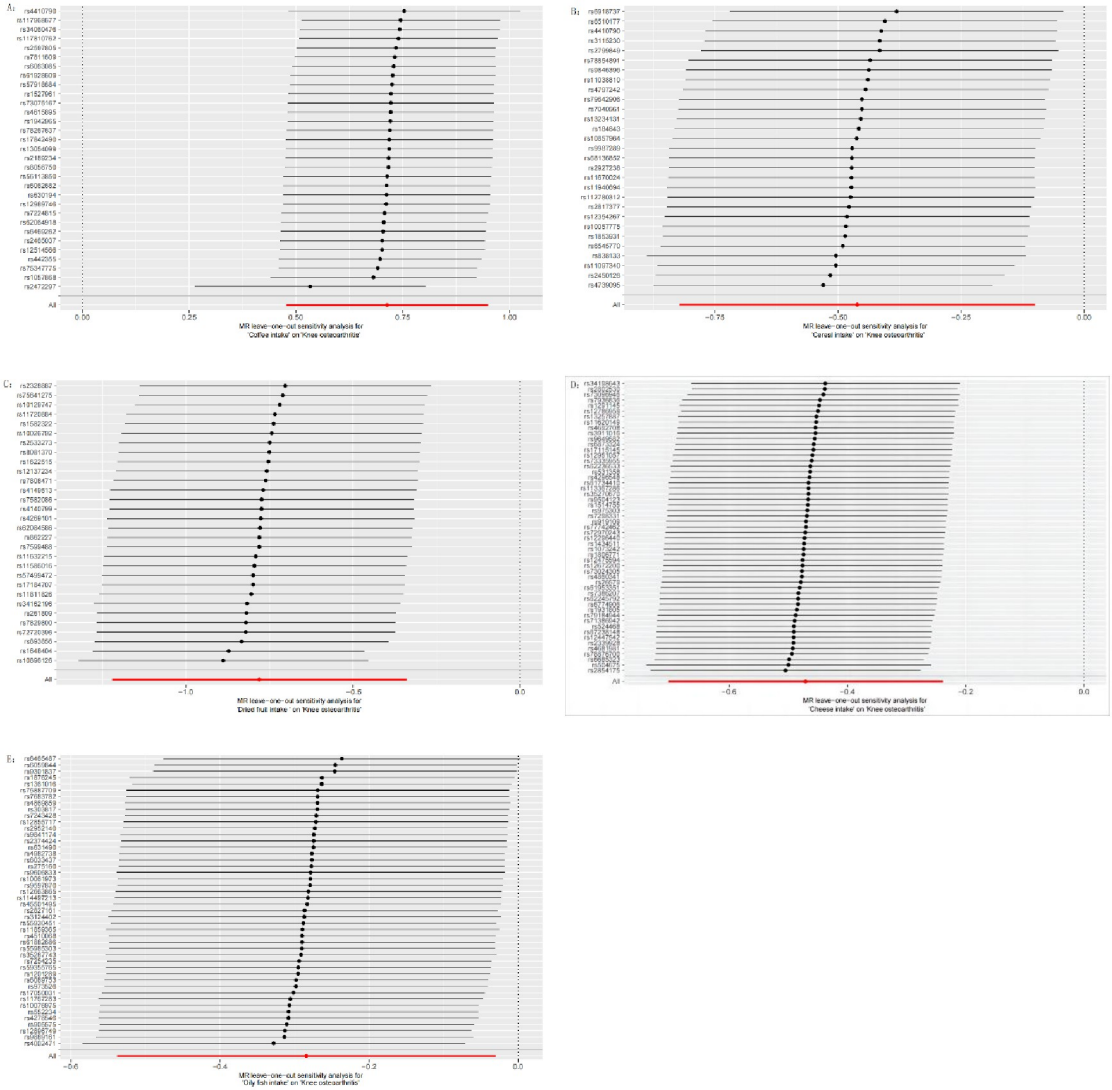
Leave-one-out sensitivity analysis between diet and KOA. Figures A, B, C, D, and E show the leave-one-out sensitivity analysis between coffee intake, cereal intake, dried fruit intake, cheese intake, and oily fish intake and KOA, respectively. Red lines represent estimates from IVW tests. IVW: Inverse Variance Weighted.

To assess heterogeneity, we performed MR-Egger regression and IVW analysis, which detected some heterogeneity across the studies (S1 File). Therefore, we used random effects IVW as the main analytical method. The MR-Egger intercept did not indicate horizontal pleiotropy (Fig 3). We also applied the leave-one-out method to exclude single nucleotide polymorphisms (SNPs) one by one and examine whether the causal association was driven by a single IV. The results confirmed the robustness of the MR analysis (Fig 4). Moreover, we present the sensitivity analysis and leave-one-out analysis results for negative results in S1 File.

## 4 Discussion

We used large-scale publicly available GWAS data to investigate the effect of genetically predicted diet on KOA risk within the MR framework. We found evidence for a causal effect of grain, dried fruit, and cheese consumption on KOA, independent of potential confounders such as obesity, BMI, hypertension, and diabetes. However, we did not observe any causal associations between KOA and processed meat, poultry, beef, non-oily fish, pork, and lamb, alcohol intake frequency, alcoholic drinks per week, tea consumption, bread intake, cooked vegetable intake, salad/raw vegetable intake, or fresh fruit intake. Moreover, MVMR analyses suggest that coffee and oily fish intake may influence KOA through obesity, BMI, hypertension, diabetes, and prolonged standing.

Many recent studies have used MR methods to explore risk or protective factors for KOA [32–34]. However, few studies have examined the association between diet and KOA using MR. Diet is related to various chronic diseases and may contribute to global morbidity and mortality [35]. A recent review study indicated that adhering to a Mediterranean or plant-based diet can reduce musculoskeletal pain [36]. A growing evidence base suggests that dietary consumption of alcohol [37], coffee [8], dried fruits [38], and grains [10] may be associated with the onset or progression of KOA. KOA affects the quality of life of patients and imposes an economic burden on the country. The findings of this study may help clinical practitioners educate patients with KOA about healthy eating. For instance, patients with signs of KOA or who already have KOA can be advised to reduce coffee intake, lose weight, control hypertension and diabetes, and increase the intake of dried fruits, grains, or cheese. Modifying dietary habits can reduce the chance of KOA recurrence and alleviate both patient discomfort and the pressure on national health insurance. Therefore, this study aims to explore the causal association between dietary factors and KOA risk.

Our study found a positive association between coffee consumption and KOA risk, which is in line with a recent MR study [39]. Several observational studies also reported a higher prevalence of KOA with increased coffee intake [8]. Our results became negative after adjusting for multiple confounders, suggesting that the risk of coffee intake on KOA may be mediated by obesity, BMI, diabetes, and hypertension. This may be attributed to the bioactive compounds in coffee, such as polyphenols, purines, and nicotinic acid, that can impair chondrocyte function and cartilage integrity. Moreover, caffeine can stimulate the nervous and endocrine systems, resulting in vasoconstriction and inflammation [40, 41]. These effects may aggravate pressure, ischemia and injury of the knee joint, thereby enhancing the risk of KOA [42].

Our MR study found that intake of cereal, dried fruit, oily fish, and cheese was associated with a lower risk of KOA. These findings are consistent with some recent observational studies that reported inverse associations of KOA risk with higher consumption of cereal [10], dried fruit [38], fish [38], and cheese [43]. However, after MVMR correction, the association between oily fish intake and KOA became negative. This suggests that the protective effect of oily fish intake on KOA may be mediated by obesity, BMI, diabetes, and hypertension. Dried fruits, cereals, and cheese are rich in calcium, an important mineral for maintaining bone health and density; adequate calcium intake may prevent and slow down the development of osteoporosis and fractures, and thus lower the risk of KOA [44]. On the other hand, oily fish contains omega-3 fatty acids, which have anti-inflammatory and immunomodulatory effects and can inhibit the degradation of joint cartilage and the release of inflammatory factors, thereby alleviating the symptoms and progression of KOA [45]. In conclusion, different foods may have different mechanisms to reduce KOA, but the underlying mechanisms are still unclear and warrant further research.

This study has several strengths. We used a two-sample MR design to explore a more comprehensive causal relationship between dietary intake and KOA, and we performed multivariate correction to ensure that the results were not confounded by other factors. We used instrumental variables with strengths much higher than 10, minimizing bias from sample overlap [46]. We also conducted multiple sensitivity analyses to assess the robustness of the study. However, some limitations should be acknowledged. We could not assign a single food to be consumed by the participants and could not avoid the interference of mixed foods. Moreover, we used data from public databases and could not stratify the analysis by sex among KOA patients. All subjects in the GWAS data were of European descent, and further research is needed to determine whether the findings can be generalized to other populations.

## 5 Conclusion

Using a two-sample UVMR design, we found that coffee intake was positively associated with KOA risk, while dried fruit, cereal, cheese, and oily fish intake were inversely associated with KOA risk. However, in the MVMR analysis, the associations of coffee and oily fish intake with KOA became negative, suggesting that obesity, BMI, diabetes, hypertension, and prolonged standing may mediate the effects of coffee and oily fish intake on KOA. Moreover, we did not find any association between other dietary intake and KOA risk.

## Supporting information

S1 File (DOC)
