# Supplementary material for: Causal relationship between diet and knee osteoarthritis: A Mendelian randomization analysis
Source: PLoS One. 2024 Jan 31;19(1):e0297269. doi: 10.1371/journal.pone.0297269 (PMC10830039; doi:10.1371/journal.pone.0297269)
Supplement: S1 File — (DOC) [file pone.0297269.s001.doc]

**Supporting information**

**Causal relationship between diet and knee osteoarthritis: a Mendelian randomization analysis**

S1 Table. Instrumental variables for the analysis of diet and knee osteoarthritis.

S2 Table. Negative results of MR analysis of the causal relationship between diet and knee osteoarthritis.

S3 Table. Heterogeneity and pleiotropy of results in the analysis of the causal relationship between diet and knee osteoarthritis.

S1 Fig. Scatter plot of the causal relationship between diet and knee osteoarthritis.

S2 Fig. Leave-one-out analysis of the negative results of diet and knee osteoarthritis.

S1 Table:

Instrumental Variable

| **1.Alcoholic drinks per week- KOA** | | | | | | | | |
| --- | --- | --- | --- | --- | --- | --- | --- | --- |
| SNP | Chr | EA | OA | Beta | EAF | SE | P | F |
| rs10085696 | 7 | G | A | -0.01605 | 0.201 | 0.00249471 | 1.24E-10 | 41.40 |
| rs11860773 | 16 | C | T | -0.01501 | 0.176 | 0.00244381 | 8.35E-10 | 37.70 |
| rs1229984 | 4 | C | T | 0.188115 | 0.953 | 0.00617852 | 1.00E-200 | 927.00 |
| rs13332432 | 16 | G | C | 0.014005 | 0.296 | 0.00214072 | 5.94E-11 | 42.80 |
| rs1387766 | 12 | A | G | -0.01083 | 0.622 | 0.00198336 | 4.79E-08 | 29.80 |
| rs16854020 | 4 | A | G | 0.018083 | 0.127 | 0.0029068 | 4.82E-10 | 38.70 |
| rs17542254 | 11 | G | A | 0.013142 | 0.251 | 0.00214605 | 8.96E-10 | 37.50 |
| rs2299409 | 7 | A | G | -0.01055 | 0.493 | 0.00193346 | 4.80E-08 | 29.80 |
| rs28601761 | 8 | G | C | 0.011299 | 0.405 | 0.00195507 | 7.60E-09 | 33.40 |
| rs28680958 | 1 | A | G | -0.01358 | 0.23 | 0.0023684 | 9.78E-09 | 32.90 |
| rs28712821 | 4 | A | G | 0.028334 | 0.594 | 0.00197411 | 1.10E-46 | 206.00 |
| rs28732378 | 3 | G | A | -0.01673 | 0.729 | 0.00219076 | 2.24E-14 | 58.30 |
| rs331939 | 4 | A | G | -0.0119 | 0.339 | 0.00202921 | 4.50E-09 | 34.40 |
| rs34121753 | 17 | G | A | 0.011069 | 0.532 | 0.00195072 | 1.39E-08 | 32.20 |
| rs4309187 | 11 | C | A | 0.014791 | 0.697 | 0.00208756 | 1.37E-12 | 50.20 |
| rs494904 | 2 | C | T | 0.015085 | 0.429 | 0.00196055 | 1.41E-14 | 59.20 |
| rs55872084 | 5 | T | G | 0.012731 | 0.218 | 0.0022684 | 1.98E-08 | 31.50 |
| rs55932213 | 9 | G | A | 0.012477 | 0.701 | 0.002216 | 1.80E-08 | 31.70 |
| rs6106989 | 20 | A | G | 0.010899 | 0.628 | 0.00198336 | 3.81E-08 | 30.20 |
| rs6739804 | 2 | C | T | -0.01297 | 0.66 | 0.00208202 | 4.72E-10 | 38.80 |
| rs676388 | 19 | C | T | 0.015098 | 0.494 | 0.00193147 | 5.49E-15 | 61.10 |
| rs6969458 | 7 | A | G | 0.012706 | 0.459 | 0.00193545 | 5.20E-11 | 43.10 |
| rs75120545 | 2 | T | C | -0.03279 | 0.022 | 0.00567307 | 7.59E-09 | 33.40 |
| rs76640332 | 17 | A | G | -0.02101 | 0.204 | 0.00238944 | 1.47E-18 | 77.30 |
| rs78234152 | 4 | A | G | 0.027654 | 0.0986 | 0.00307075 | 2.18E-19 | 81.10 |
| rs79616692 | 16 | C | G | 0.018809 | 0.11 | 0.00315231 | 2.38E-09 | 35.60 |
| rs962961 | 14 | T | C | -0.01219 | 0.329 | 0.00205146 | 2.78E-09 | 35.30 |
| **2.Alcohol intake frequency- KOA** | | | | | | | | |
| SNP | Chr | EA | OA | Beta | EAF | SE | P | F |
| rs10188314 | 2 | T | C | -0.01979 | 0.470852 | 0.00303605 | 7.20E-11 | 42.48 |
| rs10792669 | 11 | G | A | 0.017432 | 0.505254 | 0.00304065 | 9.90E-09 | 32.87 |
| rs11223617 | 11 | A | G | 0.025091 | 0.206155 | 0.00375381 | 2.30E-11 | 44.68 |
| rs11700855 | 21 | G | A | -0.0298 | 0.093465 | 0.00523292 | 1.20E-08 | 32.42 |
| rs11750777 | 5 | A | G | -0.02049 | 0.209454 | 0.00372613 | 3.80E-08 | 30.25 |
| rs117799466 | 15 | C | G | -0.01967 | 0.336989 | 0.00331968 | 3.10E-09 | 35.11 |
| rs11787216 | 8 | T | C | 0.024416 | 0.369127 | 0.00320078 | 2.40E-14 | 58.19 |
| rs11940694 | 4 | G | A | -0.04371 | 0.604193 | 0.00311609 | 1.00E-44 | 196.80 |
| rs12153855 | 6 | C | T | 0.029444 | 0.10497 | 0.00493484 | 2.40E-09 | 35.60 |
| rs12312693 | 12 | C | T | -0.01768 | 0.451772 | 0.00305026 | 6.80E-09 | 33.60 |
| rs13102973 | 4 | C | T | -0.01941 | 0.61881 | 0.00311875 | 4.90E-10 | 38.72 |
| rs13178443 | 5 | T | C | -0.01865 | 0.276349 | 0.00338983 | 3.80E-08 | 30.27 |
| rs13390019 | 2 | C | T | 0.029612 | 0.134041 | 0.00449182 | 4.30E-11 | 43.46 |
| rs1515591 | 3 | G | T | 0.01823 | 0.383186 | 0.00311635 | 4.90E-09 | 34.22 |
| rs1666658 | 11 | C | T | 0.017967 | 0.392206 | 0.00309866 | 6.70E-09 | 33.62 |
| rs17662759 | 2 | C | T | 0.030135 | 0.089115 | 0.00546031 | 3.40E-08 | 30.46 |
| rs17690703 | 17 | T | C | 0.025034 | 0.262687 | 0.00343021 | 2.90E-13 | 53.26 |
| rs186347 | 14 | T | G | 0.017949 | 0.463343 | 0.00305071 | 4.00E-09 | 34.62 |
| rs1893659 | 18 | A | C | -0.02933 | 0.459939 | 0.00305313 | 7.60E-22 | 92.26 |
| rs1937522 | 13 | G | A | 0.016898 | 0.528054 | 0.00303207 | 2.50E-08 | 31.06 |
| rs1991083 | 2 | T | C | -0.02239 | 0.679886 | 0.00325813 | 6.30E-12 | 47.24 |
| rs2043677 | 18 | T | C | 0.026113 | 0.145599 | 0.0043272 | 1.60E-09 | 36.42 |
| rs2159935 | 4 | A | G | -0.01857 | 0.490369 | 0.00302582 | 8.30E-10 | 37.68 |
| rs2244598 | 1 | C | T | -0.01838 | 0.605114 | 0.00311907 | 3.80E-09 | 34.72 |
| rs2535911 | 14 | T | C | -0.01885 | 0.354749 | 0.00316849 | 2.70E-09 | 35.38 |
| rs2622167 | 7 | A | G | -0.01912 | 0.428653 | 0.0030674 | 4.60E-10 | 38.84 |
| rs262240 | 3 | T | C | -0.01721 | 0.468553 | 0.00303483 | 1.40E-08 | 32.15 |
| rs2717063 | 2 | A | C | -0.02037 | 0.585731 | 0.00308457 | 4.00E-11 | 43.61 |
| rs28622224 | 4 | T | C | -0.01862 | 0.280364 | 0.00336834 | 3.20E-08 | 30.56 |
| rs28787109 | 1 | A | G | 0.017811 | 0.40423 | 0.00308461 | 7.70E-09 | 33.34 |
| rs2924321 | 18 | A | G | -0.01951 | 0.539592 | 0.00305022 | 1.60E-10 | 40.93 |
| rs2977454 | 8 | G | C | -0.02592 | 0.124072 | 0.00459853 | 1.70E-08 | 31.78 |
| rs34440851 | 8 | T | C | -0.02268 | 0.157151 | 0.00415058 | 4.60E-08 | 29.87 |
| rs34473884 | 10 | A | G | -0.02036 | 0.24819 | 0.00350346 | 6.20E-09 | 33.78 |
| rs34631026 | 16 | T | C | -0.01691 | 0.446061 | 0.00304832 | 2.90E-08 | 30.78 |
| rs4241258 | 2 | T | C | 0.025064 | 0.13763 | 0.00440325 | 1.30E-08 | 32.40 |
| rs4242715 | 10 | A | G | -0.01865 | 0.680585 | 0.00324831 | 9.30E-09 | 32.98 |
| rs4417025 | 1 | A | G | -0.01884 | 0.361153 | 0.00316516 | 2.70E-09 | 35.42 |
| rs4503294 | 1 | T | C | 0.018148 | 0.565333 | 0.00307049 | 3.40E-09 | 34.93 |
| rs461599 | 5 | C | A | -0.01919 | 0.462259 | 0.00303977 | 2.70E-10 | 39.85 |
| rs4726481 | 7 | T | G | 0.021761 | 0.400576 | 0.00310188 | 2.30E-12 | 49.22 |
| rs473098 | 2 | T | C | -0.02174 | 0.557689 | 0.00304345 | 9.10E-13 | 51.03 |
| rs4916723 | 5 | C | A | 0.023948 | 0.420617 | 0.00309951 | 1.10E-14 | 59.70 |
| rs4968391 | 17 | T | G | -0.01927 | 0.674892 | 0.0032265 | 2.30E-09 | 35.67 |
| rs5022348 | 18 | T | C | 0.020264 | 0.40703 | 0.00357005 | 1.40E-08 | 32.22 |
| rs550942 | 11 | T | C | 0.022401 | 0.823865 | 0.00398884 | 2.00E-08 | 31.54 |
| rs58905411 | 12 | A | G | -0.02663 | 0.410052 | 0.00307848 | 5.10E-18 | 74.85 |
| rs6030200 | 20 | A | G | -0.01953 | 0.31415 | 0.00327075 | 2.40E-09 | 35.65 |
| rs62305780 | 4 | G | C | -0.04852 | 0.102253 | 0.00506585 | 9.90E-22 | 91.74 |
| rs62339673 | 4 | A | C | 0.018294 | 0.626705 | 0.00315406 | 6.60E-09 | 33.64 |
| rs62466318 | 7 | T | C | -0.02549 | 0.202827 | 0.0037742 | 1.40E-11 | 45.62 |
| rs650558 | 17 | T | C | 0.020736 | 0.247918 | 0.0035079 | 3.40E-09 | 34.94 |
| rs6727281 | 2 | T | C | -0.02432 | 0.184023 | 0.00391964 | 5.50E-10 | 38.50 |
| rs6943160 | 7 | C | T | 0.020627 | 0.208646 | 0.00372774 | 3.10E-08 | 30.62 |
| rs71651683 | 22 | T | C | -0.07046 | 0.0142 | 0.0127906 | 3.60E-08 | 30.35 |
| rs72769229 | 2 | T | A | -0.02314 | 0.154942 | 0.00419154 | 3.40E-08 | 30.46 |
| rs72787062 | 16 | A | G | -0.02819 | 0.162767 | 0.00410324 | 6.40E-12 | 47.22 |
| rs728538 | 16 | G | T | 0.022875 | 0.168868 | 0.00406259 | 1.80E-08 | 31.70 |
| rs7298932 | 12 | G | A | -0.02372 | 0.147849 | 0.00431166 | 3.80E-08 | 30.27 |
| rs7330939 | 13 | T | C | -0.02133 | 0.720352 | 0.0034046 | 3.70E-10 | 39.25 |
| rs74679146 | 9 | C | T | -0.03207 | 0.074515 | 0.00575757 | 2.50E-08 | 31.03 |
| rs7514579 | 1 | C | A | 0.019667 | 0.232457 | 0.00359788 | 4.60E-08 | 29.88 |
| rs76082653 | 3 | T | C | 0.046427 | 0.054327 | 0.00668673 | 3.80E-12 | 48.21 |
| rs7610856 | 3 | A | C | -0.02386 | 0.429053 | 0.00307022 | 7.70E-15 | 60.42 |
| rs780569 | 1 | A | T | 0.019803 | 0.70882 | 0.00336454 | 4.00E-09 | 34.64 |
| rs80292319 | 15 | C | T | -0.03937 | 0.057704 | 0.00649583 | 1.40E-09 | 36.74 |
| rs8043563 | 16 | C | G | 0.023365 | 0.737192 | 0.00347125 | 1.70E-11 | 45.31 |
| rs838145 | 19 | A | G | 0.021955 | 0.542982 | 0.00305549 | 6.70E-13 | 51.63 |
| rs8614 | 17 | A | C | 0.024781 | 0.182509 | 0.00392537 | 2.70E-10 | 39.85 |
| rs9349379 | 6 | G | A | -0.01935 | 0.405493 | 0.00308215 | 3.50E-10 | 39.40 |
| rs9403297 | 6 | A | G | 0.018823 | 0.372967 | 0.00313037 | 1.80E-09 | 36.16 |
| rs9648478 | 7 | A | G | 0.01686 | 0.510245 | 0.00302891 | 2.60E-08 | 30.99 |
| rs9814516 | 3 | T | G | -0.02511 | 0.237423 | 0.00355589 | 1.60E-12 | 49.87 |
| rs9829192 | 3 | T | G | 0.016932 | 0.435133 | 0.00305032 | 2.80E-08 | 30.81 |
| rs9906502 | 17 | A | G | 0.023788 | 0.176998 | 0.00396178 | 1.90E-09 | 36.05 |
| rs9912298 | 17 | C | A | 0.020589 | 0.239585 | 0.00358993 | 9.70E-09 | 32.89 |
| rs9958320 | 18 | C | T | 0.024855 | 0.153147 | 0.00427092 | 5.90E-09 | 33.87 |
| **3.Processed meat intake- KOA** | | | | | | | | |
| SNP | Chr | EA | OA | Beta | EAF | SE | P | F |
| rs10454812 | 5 | C | A | -0.01996 | 0.103032 | 0.00344207 | 6.70E-09 | 33.63 |
| rs11032380 | 11 | T | A | -0.01332 | 0.333352 | 0.00222295 | 2.10E-09 | 35.92 |
| rs11887120 | 2 | T | C | 0.011962 | 0.397683 | 0.00216043 | 3.10E-08 | 30.66 |
| rs11894162 | 2 | T | C | 0.012049 | 0.547464 | 0.00210698 | 1.10E-08 | 32.70 |
| rs2029401 | 5 | G | A | 0.014631 | 0.586212 | 0.0021292 | 6.30E-12 | 47.22 |
| rs203319 | 22 | T | C | -0.01642 | 0.204594 | 0.00260186 | 2.80E-10 | 39.84 |
| rs2873054 | 3 | C | A | 0.013996 | 0.353276 | 0.00218986 | 1.60E-10 | 40.85 |
| rs34241936 | 17 | G | A | 0.032837 | 0.037369 | 0.00575337 | 1.10E-08 | 32.57 |
| rs3762621 | 2 | T | C | -0.01499 | 0.183451 | 0.00271965 | 3.60E-08 | 30.38 |
| rs4077924 | 2 | C | T | 0.012499 | 0.701892 | 0.00228468 | 4.50E-08 | 29.93 |
| rs4778053 | 15 | G | C | 0.016467 | 0.843808 | 0.00289872 | 1.30E-08 | 32.27 |
| rs6010651 | 20 | C | A | -0.01241 | 0.379439 | 0.00216796 | 1.10E-08 | 32.75 |
| rs6484504 | 11 | C | T | 0.015473 | 0.72462 | 0.0023479 | 4.40E-11 | 43.43 |
| rs6765179 | 3 | A | G | -0.01276 | 0.309968 | 0.00226606 | 1.80E-08 | 31.71 |
| rs6786550 | 3 | C | T | 0.012187 | 0.635043 | 0.00217408 | 2.10E-08 | 31.42 |
| rs6961970 | 7 | A | C | -0.01401 | 0.244698 | 0.00244137 | 9.50E-09 | 32.95 |
| rs8096167 | 18 | C | T | -0.01459 | 0.1926 | 0.00267084 | 4.70E-08 | 29.86 |
| rs838133 | 19 | G | A | 0.019014 | 0.549369 | 0.00216555 | 1.60E-18 | 77.09 |
| rs9809856 | 3 | G | A | 0.013289 | 0.475881 | 0.00209967 | 2.50E-10 | 40.06 |
| **4.Poultry intake-KOA** | | | | | | | | |
| SNP | Chr | EA | OA | Beta | EAF | SE | P | F |
| rs1051730 | 15 | A | G | -0.01088 | 0.331381 | 0.00192936 | 1.70E-08 | 31.78 |
| rs2426440 | 20 | G | A | 0.011218 | 0.732602 | 0.00205323 | 4.70E-08 | 29.85 |
| rs2565017 | 18 | A | G | 0.010952 | 0.372697 | 0.00188211 | 5.90E-09 | 33.86 |
| rs2965200 | 19 | A | G | -0.01042 | 0.640011 | 0.00190089 | 4.20E-08 | 30.05 |
| rs7829800 | 8 | G | A | 0.011458 | 0.670759 | 0.00194283 | 3.70E-09 | 34.78 |
| rs9923768 | 16 | A | G | 0.010512 | 0.59853 | 0.00185899 | 1.60E-08 | 31.98 |
| **5.Beef intake- KOA** | | | | | | | | |
| SNP | Chr | EA | OA | Beta | EAF | SE | P | F |
| rs10959890 | 9 | C | T | -0.01267 | 0.212297 | 0.00209611 | 1.50E-09 | 36.51 |
| rs1105388 | 1 | T | C | -0.01138 | 0.300147 | 0.00187563 | 1.30E-09 | 36.78 |
| rs11165829 | 1 | G | C | -0.0102 | 0.35997 | 0.00177865 | 9.80E-09 | 32.89 |
| rs11878917 | 19 | A | G | 0.015019 | 0.109688 | 0.00274812 | 4.60E-08 | 29.87 |
| rs132901 | 22 | T | C | 0.013925 | 0.787662 | 0.00209282 | 2.90E-11 | 44.27 |
| rs1470610 | 2 | C | G | -0.01222 | 0.196185 | 0.00215779 | 1.50E-08 | 32.05 |
| rs4676964 | 3 | T | C | 0.013353 | 0.510605 | 0.00172438 | 9.60E-15 | 59.97 |
| rs62169335 | 2 | T | C | -0.00969 | 0.543241 | 0.00173523 | 2.40E-08 | 31.16 |
| rs7791463 | 7 | A | G | 0.009548 | 0.534781 | 0.00171063 | 2.40E-08 | 31.16 |
| rs784251 | 18 | T | C | -0.01034 | 0.477649 | 0.00171651 | 1.70E-09 | 36.28 |
| rs79809011 | 8 | A | G | -0.02806 | 0.029465 | 0.00508482 | 3.40E-08 | 30.45 |
| **6.Non-oily fish intake- KOA** | | | | | | | | |
| SNP | Chr | EA | OA | Beta | EAF | SE | P | F |
| rs11680516 | 2 | C | T | 0.012284 | 0.202388 | 0.00202829 | 1.40E-09 | 36.68 |
| rs16822430 | 2 | C | T | 0.011634 | 0.23321 | 0.00191996 | 1.40E-09 | 36.72 |
| rs17317920 | 7 | G | A | 0.00906 | 0.479238 | 0.0016312 | 2.80E-08 | 30.85 |
| rs35287743 | 12 | T | G | -0.01774 | 0.11583 | 0.00255244 | 3.60E-12 | 48.31 |
| rs3799077 | 6 | G | T | -0.01073 | 0.309993 | 0.00175764 | 1.00E-09 | 37.30 |
| rs4318925 | 6 | T | C | -0.01504 | 0.177245 | 0.0021207 | 1.30E-12 | 50.28 |
| rs6957745 | 7 | C | T | -0.01218 | 0.202978 | 0.00202331 | 1.80E-09 | 36.23 |
| rs7148387 | 14 | G | A | -0.00931 | 0.590715 | 0.00165022 | 1.70E-08 | 31.80 |
| rs838133 | 19 | G | A | 0.016184 | 0.549409 | 0.00167625 | 4.70E-22 | 93.22 |
| **7.Oily fish intake- KOA** | | | | | | | | |
| SNP | Chr | EA | OA | Beta | EAF | SE | P | F |
| rs10061973 | 5 | T | G | -0.01085 | 0.5139 | 0.00191598 | 1.50E-08 | 32.08 |
| rs10076975 | 5 | C | T | 0.011239 | 0.381413 | 0.0019672 | 1.10E-08 | 32.64 |
| rs114497213 | 3 | T | G | 0.027323 | 0.05481 | 0.00423605 | 1.10E-10 | 41.60 |
| rs11767283 | 7 | G | A | 0.017674 | 0.221709 | 0.00231943 | 2.50E-14 | 58.06 |
| rs11859365 | 16 | C | A | 0.022567 | 0.253765 | 0.00219679 | 9.40E-25 | 105.53 |
| rs1201289 | 3 | G | T | -0.01073 | 0.394525 | 0.0019595 | 4.40E-08 | 29.97 |
| rs12663865 | 6 | A | G | 0.012772 | 0.758185 | 0.00223239 | 1.10E-08 | 32.73 |
| rs12855717 | 13 | T | C | -0.01223 | 0.526774 | 0.00192275 | 2.00E-10 | 40.46 |
| rs12896749 | 14 | C | G | -0.01096 | 0.384721 | 0.00196607 | 2.50E-08 | 31.10 |
| rs1361016 | 13 | G | T | 0.014957 | 0.844853 | 0.00265295 | 1.70E-08 | 31.78 |
| rs17050031 | 2 | T | C | -0.01205 | 0.4801 | 0.0019207 | 3.50E-10 | 39.35 |
| rs1876245 | 3 | C | T | 0.015116 | 0.431482 | 0.00193122 | 5.00E-15 | 61.27 |
| rs2374424 | 11 | G | A | -0.01144 | 0.601525 | 0.00195609 | 4.90E-09 | 34.23 |
| rs275160 | 2 | C | T | 0.012119 | 0.700597 | 0.00210096 | 8.00E-09 | 33.27 |
| rs2827161 | 21 | G | T | 0.010711 | 0.422842 | 0.00193719 | 3.20E-08 | 30.57 |
| rs2952140 | 17 | T | C | -0.01068 | 0.482607 | 0.00191504 | 2.50E-08 | 31.09 |
| rs303817 | 12 | G | A | 0.01358 | 0.751146 | 0.00220965 | 8.00E-10 | 37.77 |
| rs3124402 | 13 | G | A | -0.022 | 0.733261 | 0.00215651 | 1.90E-24 | 104.09 |
| rs35287743 | 12 | T | G | -0.02822 | 0.115869 | 0.00301071 | 7.00E-21 | 87.86 |
| rs4002471 | 19 | T | C | -0.01924 | 0.547362 | 0.0019244 | 1.50E-23 | 100.00 |
| rs4278546 | 11 | G | A | 0.012556 | 0.441085 | 0.00193806 | 9.30E-11 | 41.97 |
| rs4510068 | 17 | T | G | -0.01301 | 0.402798 | 0.00197082 | 4.00E-11 | 43.60 |
| rs45501495 | 1 | T | C | 0.01568 | 0.236013 | 0.00225713 | 3.70E-12 | 48.26 |
| rs4869859 | 6 | C | T | 0.01401 | 0.449939 | 0.00192207 | 3.10E-13 | 53.13 |
| rs4982738 | 14 | A | G | 0.010861 | 0.582899 | 0.00196936 | 3.50E-08 | 30.41 |
| rs552234 | 9 | A | G | -0.01165 | 0.495449 | 0.00191252 | 1.10E-09 | 37.09 |
| rs55930451 | 2 | T | C | -0.01705 | 0.108001 | 0.00307567 | 2.90E-08 | 30.74 |
| rs55985303 | 2 | A | G | 0.012974 | 0.241079 | 0.00223629 | 6.60E-09 | 33.66 |
| rs59355765 | 18 | T | C | -0.01625 | 0.160079 | 0.00260966 | 4.70E-10 | 38.78 |
| rs6033437 | 20 | A | C | 0.012468 | 0.257329 | 0.00220977 | 1.70E-08 | 31.84 |
| rs6059844 | 20 | G | A | 0.011 | 0.495121 | 0.00191451 | 9.20E-09 | 33.01 |
| rs6089753 | 20 | T | C | -0.01154 | 0.530967 | 0.0019184 | 1.80E-09 | 36.20 |
| rs61882686 | 11 | A | C | 0.019756 | 0.085234 | 0.0034254 | 8.00E-09 | 33.26 |
| rs631490 | 11 | C | G | -0.01514 | 0.709106 | 0.00210253 | 6.00E-13 | 51.85 |
| rs6465487 | 7 | G | A | -0.01235 | 0.399814 | 0.00195628 | 2.70E-10 | 39.88 |
| rs7243428 | 18 | G | A | -0.01298 | 0.224648 | 0.00229212 | 1.50E-08 | 32.06 |
| rs7254235 | 19 | G | A | -0.01063 | 0.577254 | 0.00194012 | 4.30E-08 | 30.02 |
| rs75887709 | 19 | G | A | -0.01591 | 0.13588 | 0.0028148 | 1.60E-08 | 31.95 |
| rs7683782 | 4 | G | C | 0.014478 | 0.833405 | 0.00257399 | 1.90E-08 | 31.64 |
| rs905575 | 3 | G | C | 0.013882 | 0.82397 | 0.00251917 | 3.60E-08 | 30.37 |
| rs9301837 | 13 | A | C | -0.01574 | 0.143273 | 0.00273045 | 8.10E-09 | 33.24 |
| rs9597870 | 13 | G | T | -0.01274 | 0.245844 | 0.00223046 | 1.10E-08 | 32.65 |
| rs9606833 | 22 | C | T | 0.016986 | 0.243593 | 0.00223087 | 2.70E-14 | 57.97 |
| rs973526 | 1 | T | C | -0.01151 | 0.513307 | 0.00192962 | 2.50E-09 | 35.56 |
| rs9841174 | 3 | C | T | 0.014778 | 0.373913 | 0.00198025 | 8.50E-14 | 55.69 |
| rs9889161 | 16 | T | G | -0.01332 | 0.357929 | 0.0020015 | 2.80E-11 | 44.29 |
| **8.Pork intake- KOA** | | | | | | | | |
| SNP | Chr | EA | OA | Beta | EAF | SE | P | F |
| rs10972033 | 9 | T | G | 0.008976 | 0.45643 | 0.00147851 | 1.30E-09 | 36.85 |
| rs11211124 | 1 | C | T | -0.00995 | 0.230603 | 0.00175408 | 1.40E-08 | 32.19 |
| rs2387807 | 12 | T | C | -0.01508 | 0.077893 | 0.00274837 | 4.10E-08 | 30.12 |
| rs254152 | 5 | G | C | -0.01043 | 0.23498 | 0.00174217 | 2.20E-09 | 35.82 |
| rs34161520 | 10 | G | C | 0.011592 | 0.160381 | 0.0020206 | 9.60E-09 | 32.91 |
| rs36124222 | 18 | C | T | 0.008409 | 0.433239 | 0.00150083 | 2.10E-08 | 31.40 |
| rs3964074 | 16 | C | T | -0.00895 | 0.546932 | 0.00148347 | 1.60E-09 | 36.41 |
| rs4146837 | 15 | T | C | 0.008792 | 0.455561 | 0.0014944 | 4.00E-09 | 34.62 |
| rs7641973 | 3 | A | G | 0.008446 | 0.353362 | 0.00154097 | 4.20E-08 | 30.04 |
| rs838133 | 19 | G | A | 0.010894 | 0.549354 | 0.00152491 | 9.00E-13 | 51.04 |
| rs9973426 | 2 | G | A | 0.011085 | 0.176598 | 0.00193678 | 1.00E-08 | 32.76 |
| **9.Lamb/mutton intake- KOA** | | | | | | | | |
| SNP | Chr | EA | OA | Beta | EAF | SE | P | F |
| rs11090045 | 22 | A | G | -0.01068 | 0.307145 | 0.00160657 | 3.00E-11 | 44.16 |
| rs11743441 | 5 | T | G | -0.00884 | 0.574343 | 0.00148593 | 2.70E-09 | 35.41 |
| rs12634740 | 3 | G | T | -0.01009 | 0.252078 | 0.00169718 | 2.80E-09 | 35.32 |
| rs136548 | 22 | T | C | 0.009532 | 0.376668 | 0.00151227 | 2.90E-10 | 39.73 |
| rs139237013 | 2 | A | G | 0.018899 | 0.057668 | 0.00314007 | 1.80E-09 | 36.22 |
| rs1556147 | 9 | T | A | 0.009101 | 0.671626 | 0.00155908 | 5.30E-09 | 34.08 |
| rs16891982 | 5 | G | C | -0.02427 | 0.972143 | 0.00436437 | 2.70E-08 | 30.92 |
| rs17270057 | 19 | C | T | 0.012653 | 0.113347 | 0.00230897 | 4.30E-08 | 30.03 |
| rs1958801 | 14 | G | A | -0.00894 | 0.287998 | 0.00161749 | 3.20E-08 | 30.58 |
| rs2222760 | 2 | A | G | -0.00909 | 0.28089 | 0.00163727 | 2.80E-08 | 30.84 |
| rs2678900 | 2 | G | T | 0.010084 | 0.427924 | 0.00148119 | 9.90E-12 | 46.35 |
| rs2926119 | 16 | A | C | 0.008111 | 0.569417 | 0.00148179 | 4.40E-08 | 29.96 |
| rs3105056 | 13 | C | T | -0.01162 | 0.732738 | 0.00164854 | 1.80E-12 | 49.72 |
| rs35797675 | 7 | G | T | -0.01085 | 0.215928 | 0.0017934 | 1.40E-09 | 36.60 |
| rs3964074 | 16 | C | T | -0.00814 | 0.547043 | 0.00147218 | 3.20E-08 | 30.58 |
| rs4272399 | 8 | A | C | -0.00924 | 0.321491 | 0.00157567 | 4.50E-09 | 34.41 |
| rs4489752 | 11 | T | G | 0.013811 | 0.836007 | 0.00197586 | 2.80E-12 | 48.86 |
| rs55813438 | 16 | A | G | -0.01142 | 0.763165 | 0.0017352 | 4.70E-11 | 43.30 |
| rs56394517 | 1 | G | A | -0.01377 | 0.09584 | 0.00249124 | 3.20E-08 | 30.57 |
| rs62398404 | 6 | T | C | 0.012915 | 0.127211 | 0.00219431 | 4.00E-09 | 34.64 |
| rs6581296 | 12 | G | C | 0.01002 | 0.794714 | 0.00182433 | 4.00E-08 | 30.17 |
| rs660880 | 1 | A | G | -0.00903 | 0.51281 | 0.001464 | 6.80E-10 | 38.06 |
| rs673696 | 11 | T | C | 0.015821 | 0.080995 | 0.00268204 | 3.70E-09 | 34.80 |
| rs6829572 | 4 | A | G | 0.008401 | 0.456717 | 0.00147406 | 1.20E-08 | 32.48 |
| rs7447465 | 5 | C | T | 0.009582 | 0.619449 | 0.0015062 | 2.00E-10 | 40.47 |
| **10.Bread intake- KOA** | | | | | | | | |
| SNP | Chr | EA | OA | Beta | EAF | SE | P | F |
| rs10761661 | 10 | T | C | -0.01149 | 0.45322 | 0.00200486 | 1.00E-08 | 32.82 |
| rs11183201 | 12 | C | T | -0.01673 | 0.507856 | 0.00199574 | 5.30E-17 | 70.23 |
| rs11628639 | 14 | C | T | -0.0135 | 0.243333 | 0.00232226 | 6.20E-09 | 33.77 |
| rs13016665 | 2 | A | C | 0.014754 | 0.423235 | 0.00202829 | 3.50E-13 | 52.92 |
| rs13023099 | 2 | A | C | -0.01147 | 0.571784 | 0.00202287 | 1.40E-08 | 32.16 |
| rs1492988 | 2 | G | C | 0.011545 | 0.599095 | 0.00203549 | 1.40E-08 | 32.17 |
| rs17083079 | 5 | A | G | 0.030114 | 0.047423 | 0.00467938 | 1.20E-10 | 41.41 |
| rs1940033 | 11 | T | C | -0.01108 | 0.592727 | 0.00202843 | 4.70E-08 | 29.83 |
| rs2068650 | 5 | C | A | -0.01394 | 0.472074 | 0.00199929 | 3.10E-12 | 48.60 |
| rs2517678 | 6 | T | C | 0.013233 | 0.368395 | 0.00208598 | 2.20E-10 | 40.24 |
| rs28406095 | 15 | A | G | -0.01095 | 0.461729 | 0.00200012 | 4.40E-08 | 29.97 |
| rs4984685 | 16 | A | G | 0.013585 | 0.201006 | 0.00248083 | 4.40E-08 | 29.99 |
| rs55745436 | 10 | T | C | 0.013425 | 0.237279 | 0.00234263 | 1.00E-08 | 32.84 |
| rs596878 | 6 | C | A | -0.01174 | 0.449595 | 0.00201069 | 5.30E-09 | 34.09 |
| rs62091167 | 18 | C | A | -0.01384 | 0.215931 | 0.0024253 | 1.20E-08 | 32.57 |
| rs656817 | 18 | G | A | -0.01269 | 0.334442 | 0.00210929 | 1.80E-09 | 36.19 |
| rs6580721 | 12 | G | A | 0.017205 | 0.188665 | 0.00254355 | 1.30E-11 | 45.75 |
| rs73802707 | 4 | T | C | -0.01593 | 0.153694 | 0.00276165 | 8.00E-09 | 33.27 |
| rs75287965 | 2 | A | G | -0.02475 | 0.062933 | 0.00409745 | 1.50E-09 | 36.48 |
| rs7802468 | 7 | T | C | -0.02335 | 0.371501 | 0.00205629 | 6.90E-30 | 128.97 |
| rs79436018 | 7 | C | T | -0.01761 | 0.116276 | 0.00311645 | 1.60E-08 | 31.95 |
| rs9323989 | 14 | C | T | -0.01161 | 0.379169 | 0.00205547 | 1.60E-08 | 31.93 |
| rs9529024 | 13 | T | A | -0.01292 | 0.370396 | 0.00206792 | 4.20E-10 | 39.02 |
| rs9564268 | 13 | C | T | -0.01216 | 0.615619 | 0.00204932 | 3.00E-09 | 35.21 |
| rs9662365 | 1 | T | C | 0.012166 | 0.499481 | 0.00198921 | 9.60E-10 | 37.41 |
| **11.Cheese intake- KOA** | | | | | | | | |
| SNP | Chr | EA | OA | Beta | EAF | SE | P | F |
| rs1073242 | 13 | A | G | 0.015728 | 0.553825 | 0.00229156 | 6.70E-12 | 47.11 |
| rs113367286 | 7 | T | C | 0.01518 | 0.278467 | 0.00249985 | 1.30E-09 | 36.87 |
| rs11620149 | 13 | C | T | -0.01767 | 0.143308 | 0.0032085 | 3.60E-08 | 30.35 |
| rs12296440 | 12 | A | G | 0.018794 | 0.169681 | 0.00297985 | 2.80E-10 | 39.78 |
| rs12447542 | 16 | A | G | 0.019748 | 0.12558 | 0.00340698 | 6.80E-09 | 33.60 |
| rs12475594 | 2 | G | A | 0.016007 | 0.178459 | 0.00292518 | 4.40E-08 | 29.95 |
| rs12672200 | 7 | A | G | -0.01376 | 0.325794 | 0.00239456 | 9.00E-09 | 33.04 |
| rs12786959 | 11 | T | A | -0.01607 | 0.196267 | 0.00281988 | 1.20E-08 | 32.48 |
| rs1291145 | 20 | C | T | -0.02025 | 0.685848 | 0.00241026 | 4.40E-17 | 70.58 |
| rs12951057 | 17 | G | C | -0.02116 | 0.165627 | 0.00304376 | 3.60E-12 | 48.31 |
| rs13257887 | 8 | C | T | 0.016181 | 0.358942 | 0.00256186 | 2.70E-10 | 39.89 |
| rs1434511 | 18 | T | C | 0.012965 | 0.455286 | 0.00225868 | 9.50E-09 | 32.95 |
| rs1514755 | 2 | G | A | 0.016375 | 0.239596 | 0.00261651 | 3.90E-10 | 39.17 |
| rs17115145 | 14 | T | C | -0.01288 | 0.401282 | 0.00228956 | 1.80E-08 | 31.65 |
| rs1806771 | 10 | G | T | -0.02215 | 0.087876 | 0.004035 | 4.10E-08 | 30.13 |
| rs1931805 | 6 | C | T | 0.012636 | 0.500062 | 0.00223591 | 1.60E-08 | 31.94 |
| rs2339928 | 2 | A | G | 0.014858 | 0.704069 | 0.00244494 | 1.20E-09 | 36.93 |
| rs26579 | 5 | C | G | -0.0128 | 0.586217 | 0.00229427 | 2.40E-08 | 31.13 |
| rs2802530 | 1 | A | G | 0.018627 | 0.876502 | 0.00339704 | 4.20E-08 | 30.07 |
| rs2854175 | 17 | A | C | 0.016994 | 0.257474 | 0.00256857 | 3.70E-11 | 43.77 |
| rs34198643 | 7 | T | C | -0.0167 | 0.224165 | 0.00267855 | 4.50E-10 | 38.87 |
| rs35270670 | 15 | G | A | 0.016379 | 0.217962 | 0.00270974 | 1.50E-09 | 36.54 |
| rs3911016 | 9 | G | T | 0.021357 | 0.120938 | 0.0034399 | 5.30E-10 | 38.55 |
| rs4296548 | 3 | G | T | 0.013025 | 0.609594 | 0.00228781 | 1.20E-08 | 32.41 |
| rs4681981 | 3 | A | C | -0.01244 | 0.469086 | 0.00224121 | 2.90E-08 | 30.79 |
| rs4692708 | 4 | C | A | 0.01473 | 0.252659 | 0.00258904 | 1.30E-08 | 32.37 |
| rs4860341 | 4 | C | T | 0.024365 | 0.928742 | 0.00435152 | 2.20E-08 | 31.35 |
| rs504675 | 2 | T | C | 0.027442 | 0.352634 | 0.00234182 | 1.00E-31 | 137.32 |
| rs524468 | 12 | G | A | -0.01424 | 0.260633 | 0.00255161 | 2.40E-08 | 31.15 |
| rs531358 | 1 | T | C | 0.013167 | 0.649768 | 0.00233731 | 1.80E-08 | 31.73 |
| rs61734410 | 16 | T | C | 0.016654 | 0.255213 | 0.00262279 | 2.20E-10 | 40.32 |
| rs61953351 | 12 | T | G | 0.014593 | 0.250366 | 0.00257996 | 1.50E-08 | 31.99 |
| rs62236533 | 22 | A | G | 0.024762 | 0.108791 | 0.00364674 | 1.10E-11 | 46.11 |
| rs62245792 | 3 | A | T | -0.01793 | 0.150049 | 0.00316317 | 1.40E-08 | 32.15 |
| rs6685323 | 1 | T | C | -0.01319 | 0.309293 | 0.00241592 | 4.80E-08 | 29.80 |
| rs67238148 | 11 | T | G | 0.016543 | 0.217471 | 0.00271432 | 1.10E-09 | 37.15 |
| rs6774906 | 3 | C | A | 0.031629 | 0.040653 | 0.00567262 | 2.50E-08 | 31.09 |
| rs6873324 | 5 | C | A | -0.01248 | 0.425802 | 0.00227073 | 3.90E-08 | 30.19 |
| rs71386942 | 16 | A | C | 0.014455 | 0.268947 | 0.00252205 | 9.90E-09 | 32.85 |
| rs72970243 | 2 | A | G | 0.022201 | 0.12044 | 0.00340166 | 6.70E-11 | 42.60 |
| rs7298331 | 12 | C | A | -0.01318 | 0.604531 | 0.00230588 | 1.10E-08 | 32.65 |
| rs73024305 | 11 | C | G | 0.032537 | 0.054789 | 0.00492718 | 4.00E-11 | 43.61 |
| rs73096946 | 4 | C | T | -0.02059 | 0.157396 | 0.00306664 | 1.90E-11 | 45.09 |
| rs73335955 | 10 | C | T | 0.027773 | 0.05334 | 0.00497824 | 2.40E-08 | 31.12 |
| rs7386207 | 8 | T | C | -0.0125 | 0.563517 | 0.00226925 | 3.60E-08 | 30.33 |
| rs77742462 | 3 | G | A | -0.04747 | 0.020525 | 0.00827804 | 9.80E-09 | 32.88 |
| rs78876700 | 1 | A | G | 0.018097 | 0.137431 | 0.00327852 | 3.40E-08 | 30.47 |
| rs79184944 | 3 | A | T | 0.019602 | 0.134346 | 0.00328458 | 2.40E-09 | 35.61 |
| rs7936836 | 11 | A | C | 0.015903 | 0.417538 | 0.00227272 | 2.60E-12 | 48.96 |
| rs919109 | 17 | C | G | 0.019938 | 0.138752 | 0.00324329 | 7.90E-10 | 37.79 |
| rs9504123 | 6 | C | A | 0.014171 | 0.274743 | 0.00250387 | 1.50E-08 | 32.03 |
| rs9649582 | 7 | T | A | -0.01462 | 0.317256 | 0.00241153 | 1.40E-09 | 36.74 |
| rs975303 | 6 | G | A | 0.021276 | 0.181313 | 0.00290699 | 2.50E-13 | 53.56 |
| **12.Cooked vegetable intake- KOA** | | | | | | | | |
| SNP | Chr | EA | OA | Beta | EAF | SE | P | F |
| rs10161952 | 13 | C | A | -0.00958 | 0.31274 | 0.00168589 | 1.30E-08 | 32.29 |
| rs11138705 | 9 | C | G | 0.010372 | 0.757204 | 0.00183004 | 1.40E-08 | 32.12 |
| rs12629972 | 3 | C | T | 0.0118 | 0.588311 | 0.00159079 | 1.20E-13 | 55.02 |
| rs1816263 | 5 | C | T | 0.009581 | 0.280188 | 0.00174063 | 3.70E-08 | 30.30 |
| rs2052063 | 10 | T | C | -0.00946 | 0.515902 | 0.0015679 | 1.60E-09 | 36.38 |
| rs2102738 | 2 | C | A | -0.01216 | 0.172403 | 0.00208302 | 5.30E-09 | 34.07 |
| rs2252508 | 1 | G | A | 0.009102 | 0.480256 | 0.00156216 | 5.70E-09 | 33.95 |
| rs2844672 | 6 | A | G | -0.00964 | 0.624125 | 0.00160923 | 2.10E-09 | 35.90 |
| rs28450747 | 4 | A | G | -0.01016 | 0.23257 | 0.0018547 | 4.30E-08 | 30.01 |
| rs34155012 | 22 | T | C | 0.010558 | 0.227386 | 0.00192208 | 3.90E-08 | 30.18 |
| rs838133 | 19 | G | A | 0.011688 | 0.549575 | 0.00161465 | 4.50E-13 | 52.40 |
| **13.Tea intake- KOA** | | | | | | | | |
| SNP | Chr | EA | OA | Beta | EAF | SE | P | F |
| rs10741694 | 11 | C | T | 0.015004 | 0.627915 | 0.00219355 | 7.90E-12 | 46.78 |
| rs10752269 | 10 | A | G | -0.01287 | 0.506082 | 0.00211975 | 1.30E-09 | 36.88 |
| rs10764990 | 10 | A | G | -0.01219 | 0.607155 | 0.00216898 | 1.90E-08 | 31.59 |
| rs11164870 | 1 | G | C | -0.01196 | 0.604574 | 0.00218232 | 4.20E-08 | 30.04 |
| rs1156588 | 2 | G | A | -0.01545 | 0.210071 | 0.00260325 | 2.90E-09 | 35.24 |
| rs11587444 | 1 | G | A | 0.014033 | 0.393464 | 0.00217078 | 1.00E-10 | 41.79 |
| rs12591786 | 15 | T | C | -0.01844 | 0.158804 | 0.00294243 | 3.70E-10 | 39.27 |
| rs13282783 | 8 | T | C | -0.01358 | 0.285899 | 0.00235432 | 7.90E-09 | 33.29 |
| rs132904 | 22 | C | G | 0.016601 | 0.778651 | 0.00255257 | 7.80E-11 | 42.30 |
| rs141071726 | 7 | A | G | 0.040732 | 0.026713 | 0.00681204 | 2.20E-09 | 35.75 |
| rs1453548 | 11 | A | T | -0.01334 | 0.664929 | 0.00224973 | 3.00E-09 | 35.17 |
| rs1481012 | 4 | G | A | -0.02624 | 0.112209 | 0.00335608 | 5.30E-15 | 61.15 |
| rs149805207 | 6 | G | A | -0.07193 | 0.008538 | 0.0125823 | 1.10E-08 | 32.68 |
| rs17245213 | 11 | A | G | -0.01465 | 0.208046 | 0.00260905 | 2.00E-08 | 31.52 |
| rs17576658 | 13 | A | G | -0.01348 | 0.247081 | 0.00245655 | 4.10E-08 | 30.12 |
| rs17685 | 7 | A | G | 0.023066 | 0.277512 | 0.00236195 | 1.60E-22 | 95.36 |
| rs2117137 | 3 | G | A | 0.012995 | 0.405148 | 0.0021557 | 1.70E-09 | 36.34 |
| rs2273447 | 20 | T | A | 0.017472 | 0.203788 | 0.00263421 | 3.30E-11 | 43.99 |
| rs2279844 | 17 | A | G | -0.01199 | 0.379343 | 0.00218318 | 4.00E-08 | 30.15 |
| rs2351187 | 10 | A | G | 0.012902 | 0.318935 | 0.0022823 | 1.60E-08 | 31.96 |
| rs2478875 | 6 | G | A | 0.021894 | 0.208758 | 0.00261129 | 5.10E-17 | 70.30 |
| rs2645929 | 13 | G | A | -0.01498 | 0.813066 | 0.0027166 | 3.50E-08 | 30.42 |
| rs34619 | 5 | A | G | 0.011712 | 0.430905 | 0.0021375 | 4.30E-08 | 30.02 |
| rs4410790 | 7 | C | T | 0.040551 | 0.631224 | 0.00219507 | 3.40E-76 | 341.27 |
| rs4808193 | 19 | C | T | 0.015115 | 0.335324 | 0.0022472 | 1.70E-11 | 45.24 |
| rs4817505 | 21 | C | T | 0.015068 | 0.38998 | 0.0021746 | 4.20E-12 | 48.01 |
| rs56188862 | 1 | C | T | -0.01576 | 0.387454 | 0.0021747 | 4.30E-13 | 52.50 |
| rs56348300 | 9 | G | C | 0.015882 | 0.184619 | 0.00273191 | 6.10E-09 | 33.80 |
| rs57462170 | 3 | A | G | 0.019151 | 0.108773 | 0.00340563 | 1.90E-08 | 31.62 |
| rs57631352 | 19 | G | A | -0.0131 | 0.296859 | 0.00232117 | 1.70E-08 | 31.87 |
| rs6829 | 13 | T | C | -0.01192 | 0.596155 | 0.00216546 | 3.70E-08 | 30.28 |
| rs713598 | 7 | G | C | 0.013397 | 0.402254 | 0.00215659 | 5.20E-10 | 38.59 |
| rs72797284 | 5 | G | A | -0.01711 | 0.270797 | 0.00238353 | 7.00E-13 | 51.56 |
| rs7757102 | 6 | G | A | -0.0118 | 0.555426 | 0.00213302 | 3.10E-08 | 30.62 |
| rs9302428 | 16 | G | C | 0.012246 | 0.635799 | 0.00220122 | 2.60E-08 | 30.95 |
| rs9624470 | 22 | A | G | 0.025207 | 0.580054 | 0.00215485 | 1.30E-31 | 136.84 |
| rs9648476 | 7 | A | G | 0.012501 | 0.622954 | 0.00218542 | 1.10E-08 | 32.72 |
| rs977474 | 12 | T | C | 0.021781 | 0.833746 | 0.00285559 | 2.40E-14 | 58.18 |
| **14.Fresh fruit intake- KOA** | | | | | | | | |
| SNP | Chr | EA | OA | Beta | EAF | SE | P | F |
| rs10064431 | 5 | C | T | -0.00757 | 0.522495 | 0.00122365 | 6.00E-10 | 38.31 |
| rs10192394 | 2 | T | C | -0.00766 | 0.528785 | 0.0012287 | 4.50E-10 | 38.88 |
| rs10249294 | 7 | A | G | 0.019564 | 0.372973 | 0.00126299 | 4.10E-54 | 239.94 |
| rs10271924 | 7 | T | C | -0.00705 | 0.492595 | 0.00125592 | 2.00E-08 | 31.48 |
| rs1051547 | 16 | C | T | -0.00758 | 0.561537 | 0.00124161 | 1.10E-09 | 37.22 |
| rs10840126 | 11 | G | A | -0.00772 | 0.376121 | 0.00128576 | 1.90E-09 | 36.03 |
| rs11032362 | 11 | A | G | 0.012393 | 0.090976 | 0.00212339 | 5.30E-09 | 34.06 |
| rs11085749 | 19 | A | G | -0.00773 | 0.387076 | 0.00125477 | 7.10E-10 | 37.99 |
| rs11248509 | 10 | T | A | 0.00733 | 0.371237 | 0.00126801 | 7.40E-09 | 33.41 |
| rs11896330 | 2 | A | G | -0.00845 | 0.632839 | 0.0012744 | 3.40E-11 | 43.92 |
| rs12044599 | 1 | G | A | 0.009421 | 0.21008 | 0.0015033 | 3.70E-10 | 39.27 |
| rs12536253 | 7 | C | G | -0.00816 | 0.24901 | 0.00141599 | 8.30E-09 | 33.20 |
| rs12641371 | 4 | T | C | 0.007913 | 0.433148 | 0.00123354 | 1.40E-10 | 41.15 |
| rs12780952 | 10 | A | G | 0.007475 | 0.286382 | 0.00135361 | 3.40E-08 | 30.49 |
| rs12885598 | 14 | A | G | 0.007515 | 0.596671 | 0.00124738 | 1.70E-09 | 36.30 |
| rs13072255 | 3 | C | A | 0.00898 | 0.493966 | 0.00122355 | 2.10E-13 | 53.87 |
| rs139042899 | 17 | C | A | 0.035995 | 0.013469 | 0.00608106 | 3.20E-09 | 35.04 |
| rs149449 | 5 | A | G | 0.007289 | 0.489167 | 0.00122061 | 2.40E-09 | 35.66 |
| rs17049185 | 2 | T | G | 0.00804 | 0.267872 | 0.00139033 | 7.30E-09 | 33.44 |
| rs1866823 | 8 | A | G | 0.007431 | 0.544469 | 0.0012406 | 2.10E-09 | 35.88 |
| rs2093654 | 9 | G | A | 0.007129 | 0.388133 | 0.00125874 | 1.50E-08 | 32.08 |
| rs2143081 | 6 | A | G | 0.008321 | 0.539822 | 0.00122891 | 1.30E-11 | 45.84 |
| rs2790688 | 1 | T | C | 0.011447 | 0.154068 | 0.00169613 | 1.50E-11 | 45.54 |
| rs329274 | 7 | G | A | 0.006818 | 0.485636 | 0.00122813 | 2.80E-08 | 30.82 |
| rs34162196 | 14 | T | C | -0.01814 | 0.1008 | 0.0020297 | 4.00E-19 | 79.89 |
| rs4302893 | 9 | A | G | 0.007389 | 0.334189 | 0.00129959 | 1.30E-08 | 32.32 |
| rs4953150 | 2 | T | C | -0.00844 | 0.344088 | 0.00129268 | 6.60E-11 | 42.62 |
| rs559734 | 1 | C | G | 0.007768 | 0.711817 | 0.00136078 | 1.10E-08 | 32.59 |
| rs586346 | 6 | C | T | -0.00692 | 0.635353 | 0.00126583 | 4.50E-08 | 29.90 |
| rs60452247 | 11 | A | G | 0.00797 | 0.363079 | 0.00126932 | 3.40E-10 | 39.42 |
| rs62051554 | 16 | A | G | 0.011612 | 0.108545 | 0.00198143 | 4.60E-09 | 34.34 |
| rs6475724 | 9 | T | C | 0.007722 | 0.727296 | 0.00137406 | 1.90E-08 | 31.58 |
| rs72974263 | 2 | T | C | 0.007387 | 0.318282 | 0.00131242 | 1.80E-08 | 31.68 |
| rs739320 | 19 | C | T | -0.009 | 0.60575 | 0.00127708 | 1.90E-12 | 49.64 |
| rs7554485 | 1 | C | T | -0.00801 | 0.611539 | 0.00125414 | 1.70E-10 | 40.79 |
| rs7818437 | 8 | C | T | -0.00805 | 0.235903 | 0.00145251 | 3.00E-08 | 30.70 |
| rs78537042 | 21 | A | C | -0.01193 | 0.086801 | 0.00218432 | 4.80E-08 | 29.81 |
| rs7869969 | 9 | G | A | 0.007567 | 0.330836 | 0.00129919 | 5.70E-09 | 33.93 |
| rs7982441 | 13 | C | T | -0.00841 | 0.731883 | 0.00137614 | 9.80E-10 | 37.37 |
| rs8095324 | 18 | G | A | -0.00695 | 0.404041 | 0.00124903 | 2.70E-08 | 30.93 |
| rs817223 | 2 | C | T | -0.00727 | 0.481314 | 0.00122339 | 2.80E-09 | 35.30 |
| rs862227 | 16 | G | A | -0.01015 | 0.457858 | 0.00122398 | 1.10E-16 | 68.70 |
| rs9517948 | 13 | T | C | 0.006953 | 0.451352 | 0.00123287 | 1.70E-08 | 31.81 |
| **15.Cereal intake- KOA** | | | | | | | | |
| SNP | Chr | EA | OA | Beta | EAF | SE | P | F |
| rs10057775 | 5 | C | T | 0.020029 | 0.893563 | 0.00289378 | 4.50E-12 | 47.91 |
| rs10857964 | 1 | C | T | 0.014087 | 0.205117 | 0.0022053 | 1.70E-10 | 40.80 |
| rs11038810 | 11 | G | A | 0.011134 | 0.644091 | 0.00186325 | 2.30E-09 | 35.71 |
| rs11097340 | 4 | T | C | -0.01153 | 0.399592 | 0.00181501 | 2.10E-10 | 40.38 |
| rs112780312 | 1 | A | G | -0.01215 | 0.274969 | 0.0020189 | 1.80E-09 | 36.21 |
| rs11670024 | 19 | G | A | 0.016011 | 0.115508 | 0.00279947 | 1.10E-08 | 32.71 |
| rs11940694 | 4 | G | A | -0.01266 | 0.604068 | 0.00183426 | 5.00E-12 | 47.67 |
| rs12354267 | 1 | C | T | 0.011647 | 0.309145 | 0.00193279 | 1.70E-09 | 36.31 |
| rs13234131 | 7 | G | A | 0.017012 | 0.128369 | 0.0026602 | 1.60E-10 | 40.89 |
| rs184643 | 2 | A | G | -0.01217 | 0.56672 | 0.00180474 | 1.60E-11 | 45.44 |
| rs1853931 | 6 | A | G | -0.01134 | 0.531294 | 0.00181017 | 3.80E-10 | 39.22 |
| rs2450126 | 11 | G | A | -0.0149 | 0.156746 | 0.00245669 | 1.30E-09 | 36.79 |
| rs2799849 | 9 | T | C | -0.01233 | 0.678123 | 0.00190573 | 9.80E-11 | 41.85 |
| rs2817377 | 6 | A | G | 0.009901 | 0.537948 | 0.00178933 | 3.10E-08 | 30.62 |
| rs2927238 | 8 | G | T | 0.010247 | 0.613181 | 0.0018288 | 2.10E-08 | 31.39 |
| rs3115230 | 4 | A | C | -0.01148 | 0.752001 | 0.00207131 | 3.00E-08 | 30.71 |
| rs4410790 | 7 | C | T | -0.01091 | 0.630668 | 0.00184697 | 3.40E-09 | 34.92 |
| rs4739095 | 8 | A | G | -0.01287 | 0.765732 | 0.00210523 | 9.90E-10 | 37.35 |
| rs4797242 | 18 | A | C | 0.011431 | 0.297207 | 0.00194889 | 4.50E-09 | 34.40 |
| rs6510177 | 19 | C | T | -0.01304 | 0.805589 | 0.00228744 | 1.20E-08 | 32.48 |
| rs6545770 | 2 | T | A | -0.01373 | 0.748101 | 0.00206041 | 2.70E-11 | 44.39 |
| rs68136852 | 17 | A | C | -0.01412 | 0.152389 | 0.00247848 | 1.20E-08 | 32.47 |
| rs6918737 | 6 | A | T | 0.013733 | 0.234488 | 0.00210976 | 7.60E-11 | 42.37 |
| rs7040561 | 9 | A | T | -0.01627 | 0.850635 | 0.00252002 | 1.10E-10 | 41.68 |
| rs78854891 | 19 | C | T | 0.022141 | 0.065728 | 0.00363312 | 1.10E-09 | 37.14 |
| rs79642906 | 5 | A | G | -0.01816 | 0.083314 | 0.00322812 | 1.90E-08 | 31.64 |
| rs838133 | 19 | G | A | -0.02065 | 0.549201 | 0.00184331 | 3.90E-29 | 125.52 |
| rs9846396 | 3 | T | C | 0.011967 | 0.441556 | 0.00180031 | 3.00E-11 | 44.19 |
| rs9987289 | 8 | G | A | 0.017868 | 0.908767 | 0.0030947 | 7.80E-09 | 33.34 |
| **16.Salad/raw vegetable intake- KOA** | | | | | | | | |
| SNP | Chr | EA | OA | Beta | EAF | SE | P | F |
| rs10819082 | 9 | A | G | -0.00916 | 0.66734 | 0.00151342 | 1.40E-09 | 36.67 |
| rs12203592 | 6 | T | C | -0.01029 | 0.219338 | 0.00169411 | 1.30E-09 | 36.87 |
| rs12908495 | 15 | A | C | -0.00935 | 0.242533 | 0.00166573 | 2.00E-08 | 31.51 |
| rs17460017 | 5 | T | A | 0.011174 | 0.190124 | 0.00181338 | 7.20E-10 | 37.97 |
| rs1890012 | 13 | G | T | -0.01043 | 0.19473 | 0.00180794 | 8.10E-09 | 33.25 |
| rs3095337 | 6 | C | G | -0.01262 | 0.203909 | 0.00176637 | 9.00E-13 | 51.06 |
| rs34186148 | 17 | C | G | -0.00805 | 0.370054 | 0.0014749 | 4.80E-08 | 29.80 |
| rs4083969 | 1 | G | C | 0.017133 | 0.057217 | 0.00311517 | 3.80E-08 | 30.25 |
| rs4291983 | 18 | A | C | -0.0084 | 0.517576 | 0.00142481 | 3.70E-09 | 34.76 |
| rs57221424 | 7 | G | C | 0.008941 | 0.321733 | 0.00153327 | 5.50E-09 | 34.00 |
| rs62461186 | 7 | C | A | -0.01134 | 0.179827 | 0.00185732 | 1.00E-09 | 37.30 |
| rs75248709 | 6 | T | C | -0.01973 | 0.045947 | 0.00352774 | 2.20E-08 | 31.29 |
| rs7821179 | 8 | C | G | -0.01082 | 0.846577 | 0.00197644 | 4.40E-08 | 29.96 |
| rs8130508 | 21 | A | G | 0.00874 | 0.289686 | 0.00157797 | 3.00E-08 | 30.68 |
| **17.Coffee intake- KOA** | | | | | | | | |
| SNP | Chr | EA | OA | Beta | EAF | SE | P | F |
| rs1057868 | 7 | T | C | 0.019951 | 0.284986 | 0.00178517 | 5.40E-29 | 124.90 |
| rs117810762 | 10 | A | G | 0.035909 | 0.017881 | 0.00617871 | 6.20E-09 | 33.78 |
| rs117968677 | 15 | A | G | -0.03103 | 0.024207 | 0.00551601 | 1.90E-08 | 31.65 |
| rs12514566 | 5 | A | G | -0.0114 | 0.337107 | 0.00170562 | 2.40E-11 | 44.65 |
| rs12989746 | 2 | T | G | 0.01035 | 0.249928 | 0.00186429 | 2.80E-08 | 30.82 |
| rs13054099 | 22 | C | T | -0.01078 | 0.261004 | 0.00183597 | 4.30E-09 | 34.46 |
| rs1527961 | 2 | C | T | -0.01334 | 0.1349 | 0.00236585 | 1.70E-08 | 31.81 |
| rs17842490 | 22 | G | A | -0.04517 | 0.014248 | 0.00680848 | 3.30E-11 | 44.01 |
| rs1942965 | 18 | C | T | -0.0089 | 0.504585 | 0.00161917 | 3.80E-08 | 30.24 |
| rs2189234 | 4 | G | T | 0.009987 | 0.617795 | 0.00166052 | 1.80E-09 | 36.17 |
| rs2465037 | 6 | A | C | -0.01063 | 0.343017 | 0.00170743 | 4.80E-10 | 38.77 |
| rs2472297 | 15 | T | C | 0.046471 | 0.262883 | 0.00182733 | 1.10E-142 | 646.73 |
| rs2597805 | 4 | T | C | 0.009855 | 0.682463 | 0.00175623 | 2.00E-08 | 31.49 |
| rs34060476 | 7 | G | A | 0.018429 | 0.133855 | 0.00237033 | 7.50E-15 | 60.45 |
| rs4410790 | 7 | C | T | 0.039072 | 0.632141 | 0.00167288 | 1.20E-120 | 545.51 |
| rs442355 | 8 | C | G | -0.01114 | 0.254435 | 0.00185374 | 1.90E-09 | 36.10 |
| rs4615895 | 1 | A | G | 0.012203 | 0.740926 | 0.00184972 | 4.20E-11 | 43.52 |
| rs56113850 | 19 | C | T | 0.012667 | 0.578109 | 0.00163351 | 8.90E-15 | 60.13 |
| rs57918684 | 17 | A | G | 0.012886 | 0.154747 | 0.00223845 | 8.60E-09 | 33.14 |
| rs6062682 | 20 | T | C | 0.01037 | 0.464546 | 0.00163929 | 2.50E-10 | 40.02 |
| rs6063085 | 20 | C | A | 0.010411 | 0.373473 | 0.00166919 | 4.50E-10 | 38.90 |
| rs61928609 | 12 | C | A | -0.01473 | 0.835328 | 0.00217536 | 1.30E-11 | 45.85 |
| rs62064918 | 17 | T | C | -0.01031 | 0.244545 | 0.00187872 | 4.10E-08 | 30.10 |
| rs630194 | 18 | C | T | -0.01135 | 0.343374 | 0.00169853 | 2.30E-11 | 44.68 |
| rs6469262 | 8 | C | T | -0.00915 | 0.564966 | 0.00162895 | 1.90E-08 | 31.58 |
| rs7224815 | 17 | T | A | -0.01086 | 0.407832 | 0.00164162 | 3.70E-11 | 43.77 |
| rs73075167 | 7 | T | A | -0.01606 | 0.12918 | 0.00244429 | 5.00E-11 | 43.19 |
| rs75347775 | 19 | A | G | 0.01045 | 0.244531 | 0.00187898 | 2.70E-08 | 30.93 |
| rs7811609 | 7 | T | C | 0.009139 | 0.374746 | 0.00166468 | 4.00E-08 | 30.14 |
| rs78267637 | 8 | G | C | -0.02543 | 0.038115 | 0.00431659 | 3.90E-09 | 34.70 |
| rs8056750 | 16 | T | C | 0.010533 | 0.359129 | 0.00173692 | 1.30E-09 | 36.78 |
| **18.Dried fruit intake- KOA** | | | | | | | | |
| SNP | Chr | EA | OA | Beta | EAF | SE | P | F |
| rs10026792 | 4 | A | G | 0.010847 | 0.290404 | 0.0018423 | 3.90E-09 | 34.66 |
| rs10129747 | 14 | G | A | 0.009359 | 0.530254 | 0.00168126 | 2.60E-08 | 30.99 |
| rs10896126 | 11 | G | A | -0.01501 | 0.303582 | 0.00181919 | 1.60E-16 | 68.07 |
| rs11586016 | 1 | C | G | 0.009878 | 0.371004 | 0.00173034 | 1.10E-08 | 32.59 |
| rs11632215 | 15 | C | A | -0.01414 | 0.120179 | 0.00258382 | 4.40E-08 | 29.96 |
| rs11720884 | 3 | G | A | 0.01118 | 0.250137 | 0.00193554 | 7.60E-09 | 33.36 |
| rs11811826 | 1 | A | T | 0.013218 | 0.224231 | 0.0020058 | 4.40E-11 | 43.43 |
| rs12137234 | 1 | T | C | 0.010205 | 0.303772 | 0.00183744 | 2.80E-08 | 30.85 |
| rs1582322 | 16 | G | A | 0.009943 | 0.604805 | 0.00171571 | 6.80E-09 | 33.59 |
| rs1622515 | 11 | G | A | 0.009917 | 0.484704 | 0.00167077 | 2.90E-09 | 35.23 |
| rs1648404 | 4 | T | C | 0.009416 | 0.476112 | 0.00167357 | 1.80E-08 | 31.65 |
| rs17184707 | 2 | T | C | -0.01144 | 0.212811 | 0.00204011 | 2.10E-08 | 31.43 |
| rs2328887 | 6 | C | T | 0.018948 | 0.899467 | 0.00277607 | 8.80E-12 | 46.59 |
| rs2533273 | 7 | A | C | -0.00988 | 0.48453 | 0.00167714 | 3.90E-09 | 34.68 |
| rs261809 | 1 | G | A | -0.00963 | 0.540636 | 0.00167902 | 9.80E-09 | 32.89 |
| rs34162196 | 14 | T | C | -0.02236 | 0.101001 | 0.00277168 | 7.10E-16 | 65.10 |
| rs4140799 | 14 | A | G | 0.009457 | 0.531856 | 0.00167846 | 1.80E-08 | 31.74 |
| rs4149513 | 2 | A | G | 0.01173 | 0.493537 | 0.00167139 | 2.20E-12 | 49.25 |
| rs4269101 | 3 | G | T | -0.01381 | 0.718948 | 0.00185921 | 1.10E-13 | 55.17 |
| rs57499472 | 3 | C | T | 0.009912 | 0.404131 | 0.00171869 | 8.10E-09 | 33.26 |
| rs62084586 | 17 | C | T | 0.013395 | 0.165729 | 0.00226174 | 3.20E-09 | 35.07 |
| rs72720396 | 1 | G | A | 0.011427 | 0.229157 | 0.00198541 | 8.70E-09 | 33.12 |
| rs75641275 | 1 | C | A | -0.01416 | 0.143372 | 0.00238518 | 2.90E-09 | 35.25 |
| rs7582086 | 2 | T | G | -0.00963 | 0.468273 | 0.00167381 | 8.80E-09 | 33.09 |
| rs7599488 | 2 | T | C | -0.01042 | 0.426408 | 0.00168729 | 6.70E-10 | 38.10 |
| rs7808471 | 7 | C | T | -0.01154 | 0.322136 | 0.00178604 | 1.10E-10 | 41.72 |
| rs7829800 | 8 | G | A | -0.01045 | 0.671041 | 0.00178709 | 5.10E-09 | 34.17 |
| rs8081370 | 17 | T | C | -0.01667 | 0.910232 | 0.00293795 | 1.40E-08 | 32.18 |
| rs862227 | 16 | G | A | -0.00916 | 0.458327 | 0.00167238 | 4.30E-08 | 30.03 |
| rs893856 | 10 | A | G | -0.01336 | 0.148988 | 0.00234923 | 1.30E-08 | 32.35 |

S1 Table: SNP: single nucleotide polymorphism; EA: effect allele; OA: non-effect allele; Chr: chromosome; EAF: effect allele frequency; Beta was obtained by allele-related effects; SE: standard error. Beta, SE, and P are SNP summary statistics; F: F-statistic; KOA: Knee osteoarthritis.

S2 Table:

MR analysis of negative results

| **Exposure** | **Outcome** | **Method** | **SNP (n)** | **OR (95%CI)** | **P** |
| --- | --- | --- | --- | --- | --- |
| Alcoholic drinks per week | KOA | MR Egger | 27 | 1.48 (0.99-2.20) | 0.066 |
|  |  | Weighted median | 27 | 1.29 (0.97-1.72) | 0.077 |
|  |  | IVW | 27 | 1.28 (1.00-1.65) | 0.050 |
|  |  | Simple mode | 27 | 1.43 (0.77-2.67) | 0.266 |
|  |  | Weighted mode | 27 | 1.28 (0.95-1.72) | 0.114 |
| Alcohol intake frequency | KOA | MR Egger | 77 | 0.75 (0.39-1.44) | 0.396 |
|  |  | Weighted median | 77 | 1.06 (0.89-1.27) | 0.501 |
|  |  | IVW | 77 | 1.24 (1.06-1.45) | 0.008 |
|  |  | Simple mode | 77 | 1.07 (0.67-1.70) | 0.780 |
|  |  | Weighted mode | 77 | 1.00 (0.69-1.47) | 0.985 |
| Processed meat intake | KOA | MR Egger | 19 | 0.35 (0.03-3.60) | 0.391 |
|  |  | Weighted median | 19 | 1.17 (0.71-1.94) | 0.530 |
|  |  | IVW | 19 | 1.09 (0.70-1.68) | 0.709 |
|  |  | Simple mode | 19 | 1.60 (0.64-4.01) | 0.332 |
|  |  | Weighted mode | 19 | 1.38 (0.58-3.29) | 0.479 |
| Poultry intake | KOA | MR Egger | 6 | 0.38 (1.59E-10-9.04E+08) | 0.934 |
|  |  | Weighted median | 6 | 1.32 (0.52-3.31) | 0.556 |
|  |  | IVW | 6 | 1.52 (0.74-3.12) | 0.259 |
|  |  | Simple mode | 6 | 1.28 (0.33-4.92) | 0.735 |
|  |  | Weighted mode | 6 | 1.22 (0.31-4.85) | 0.787 |
| Beef intake | KOA | MR Egger | 11 | 0.33 (0.03-4.00) | 0.410 |
|  |  | Weighted median | 11 | 0.79 (0.40-1.58) | 0.507 |
|  |  | IVW | 11 | 1.04 (0.61-1.77) | 0.881 |
|  |  | Simple mode | 11 | 0.75 (0.25-2.27) | 0.619 |
|  |  | Weighted mode | 11 | 0.73 (0.26-2.08) | 0.570 |
| Non-oily fish intake | KOA | MR Egger | 9 | 1.20 (0.07-20.04) | 0.904 |
|  |  | Weighted median | 9 | 0.84 (0.40-1.79) | 0.656 |
|  |  | IVW | 9 | 0.73 (0.40-1.31) | 0.289 |
|  |  | Simple mode | 9 | 0.92 (0.26-3.29) | 0.896 |
|  |  | Weighted mode | 9 | 0.99 (0.33-3.01) | 0.991 |
| Pork intake | KOA | MR Egger | 11 | 0.02 (2.47E-05-12.82) | 0.261 |
|  |  | Weighted median | 11 | 1.02 (0.36-2.89) | 0.971 |
|  |  | IVW | 11 | 1.66 (0.61-4.54) | 0.321 |
|  |  | Simple mode | 11 | 0.51 (0.07-3.71) | 0.522 |
|  |  | Weighted mode | 11 | 0.57 (0.12-2.62) | 0.485 |
| Lamb/mutton intake | KOA | MR Egger | 25 | 0.11 (0.01-078) | 0.037 |
|  |  | Weighted median | 25 | 1.51 (0.84-2.72) | 0.168 |
|  |  | IVW | 25 | 1.63 (1.04-2.54) | 0.032 |
|  |  | Simple mode | 25 | 1.60 (0.48-5.34) | 0.453 |
|  |  | Weighted mode | 25 | 1.58 (0.54-4.61) | 0.412 |
| Bread intake | KOA | MR Egger | 25 | 0.63 (0.13-3.16) | 0.584 |
|  |  | Weighted median | 25 | 1.09 (0.71-1.67) | 0.708 |
|  |  | IVW | 25 | 1.05 (0.72-1.53) | 0.818 |
|  |  | Simple mode | 25 | 1.11 (0.46-2.65) | 0.818 |
|  |  | Weighted mode | 25 | 1.15 (0.63-2.10) | 0.643 |
| Cooked vegetable intake | KOA | MR Egger | 11 | 4.54 (0.01-1817.85) | 0.632 |
|  |  | Weighted median | 11 | 1.36 (0.61-3.04) | 0.450 |
|  |  | IVW | 11 | 1.50 (0.84-2.68) | 0.172 |
|  |  | Simple mode | 11 | 1.08 (0.27-4.26) | 0.913 |
|  |  | Weighted mode | 11 | 1.15 (0.29-4.57) | 0.846 |
| Tea intake | KOA | MR Egger | 38 | 1.81 (0.87-3.77) | 0.121 |
|  |  | Weighted median | 38 | 1.57 (1.14-2.16) | 0.006 |
|  |  | IVW | 38 | 1.25 (0.96-1.63) | 0.094 |
|  |  | Simple mode | 38 | 1.27 (0.54-3.01) | 0.591 |
|  |  | Weighted mode | 38 | 1.65 (1.07-2.55) | 0.029 |
| Fresh fruit intake | KOA | MR Egger | 43 | 0.73 (0.18-3.01) | 0.664 |
|  |  | Weighted median | 43 | 0.82 (0.47-1.43) | 0.484 |
|  |  | IVW | 43 | 0.92 (0.61-1.38) | 0.684 |
|  |  | Simple mode | 43 | 0.70 (0.25-1.96) | 0.502 |
|  |  | Weighted mode | 43 | 0.77 (0.35-1.71) | 0.527 |
| Salad / raw vegetable intake | KOA | MR Egger | 14 | 0.13 (0.00-4.67) | 0.287 |
|  |  | Weighted median | 14 | 0.80 (0.32-2.02) | 0.644 |
|  |  | IVW | 14 | 0.71 (0.34-1.49) | 0.365 |
|  |  | Simple mode | 14 | 1.01 (0.17-5.90) | 0.989 |
|  |  | Weighted mode | 14 | 0.95 (0.14-6.44) | 0.956 |

S2 Table: MR analysis of negative results. KOA: Knee osteoarthritis, IVW: Inverse variance weighted.

S3 Table:

Sensitivity Analysis

| Exposure | Outcome | Heterogeneity test (MR-Egger) | | Heterogeneity test (IVW) | | | Horizontal pleiotropy test  (MR-Egger) | | |
| --- | --- | --- | --- | --- | --- | --- | --- | --- | --- |
|  |  | Cochran's Q | P | Cochran's Q | | P | Intercept | | P |
| Alcohol intake frequency | KOA | 164.92 | 1.06E-08 | | 170.19 | 3.58E-09 | | 0.011 | 0.13 |
| Processed meat intake | KOA | 30.56 | 0.02 | 32.25 | | 0.02 | 0.017 | | 0.35 |
| Dried fruit intake | KOA | 53.74 | 0.002 | 53.74 | | 0.003 | -0.0005 | | 0.97 |
| Cereal intake | KOA | 41.07 | 0.04 | 41.48 | | 0.05 | -0.006 | | 0.61 |
| Tea intake | KOA | 65.48 | 0.002 | 67.52 | | 0.005 | -0.007 | | 0.30 |
| Alcoholic drinks per week | KOA | 37.90 | 0.05 | 39.11 | | 0.05 | -0.004 | | 0.38 |
| Poultry intake | KOA | 3.45 | 0.49 | 3.47 | | 0.63 | 0.02 | | 0.91 |
| Beef intake | KOA | 4.04 | 0.91 | 4.88 | | 0.90 | 0.01 | | 0.38 |
| Non-oily fish intake | KOA | 8.76 | 0.27 | 8.91 | | 0.35 | -0.006 | | 0.73 |
| Oily fish intake | KOA | 63.88 | 0.03 | 63.94 | | 0.03 | -0.001 | | 0.84 |
| Pork intake | KOA | 21.14 | 0.01 | 25.52 | | 0.004 | 0.05 | | 0.21 |
| Lamb/mutton intake | KOA | 20.25 | 0.63 | 27.84 | | 0.27 | 0.03 | | 0.01 |
| Bread intake | KOA | 41.63 | 0.01 | 42.35 | | 0.01 | 0.007 | | 0.54 |
| Cheese intake | KOA | 85.67 | 0.001 | 86.02 | | 0.002 | 0.003 | | 0.65 |
| Cooked vegetable intake | KOA | 9.17 | 0.42 | 9.31 | | 0.50 | -0.01 | | 0.72 |
| Fresh fruit intake | KOA | 56.42 | 0.06 | 56.57 | | 0.07 | 0.002 | | 0.74 |
| Salad / raw vegetable intake | KOA | 19.57 | 0.08 | 21.03 | | 0.07 | 0.02 | | 0.36 |
| Coffee intake | KOA | 30.89 | 0.37 | 31.43 | | 0.39 | -0.003 | | 0.48 |

S3 Table: Sensitivity analysis of MR, including pleiotropy analysis and heterogeneity analysis. KOA: Knee osteoarthritis.

S1 Fig:


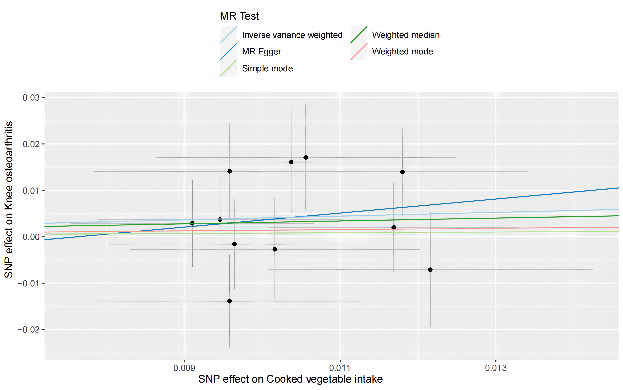

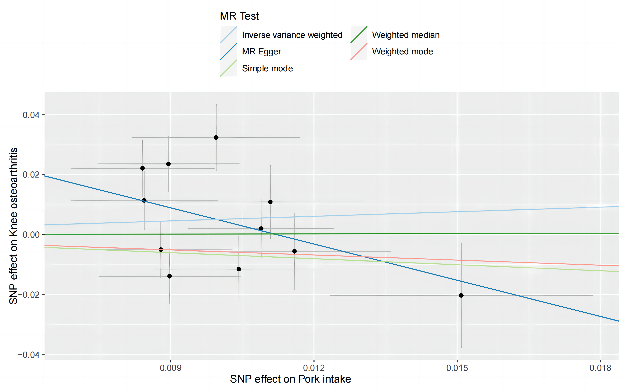

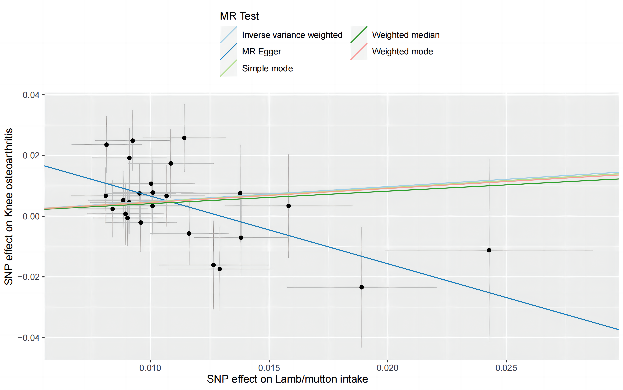

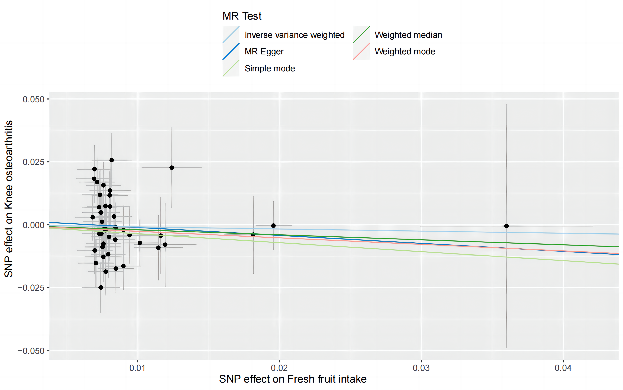

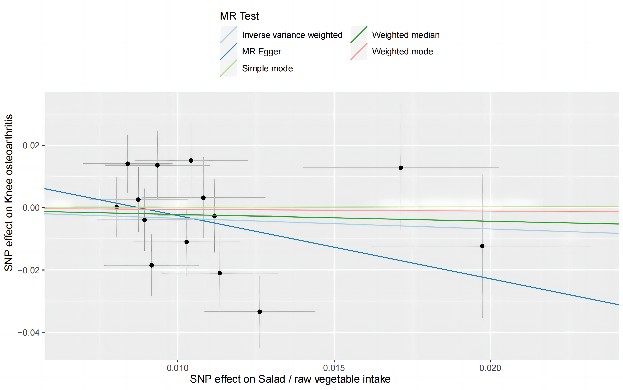

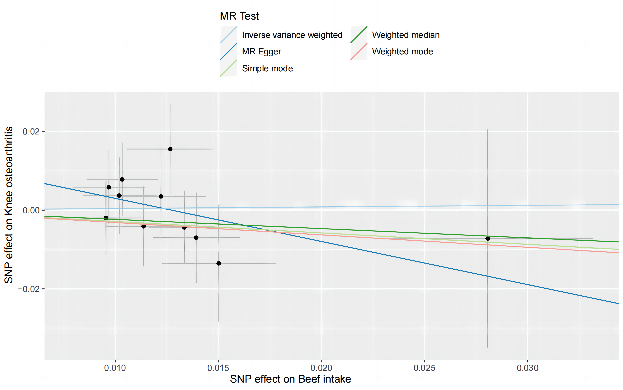

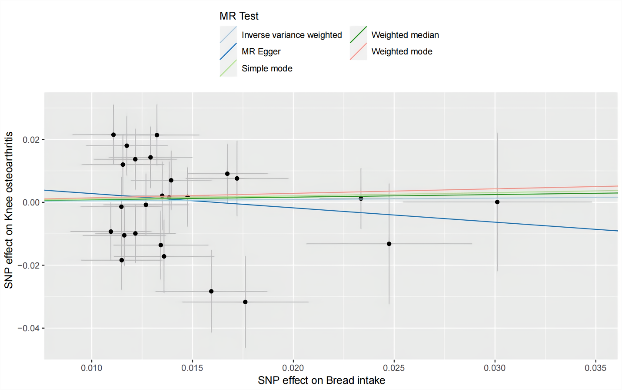

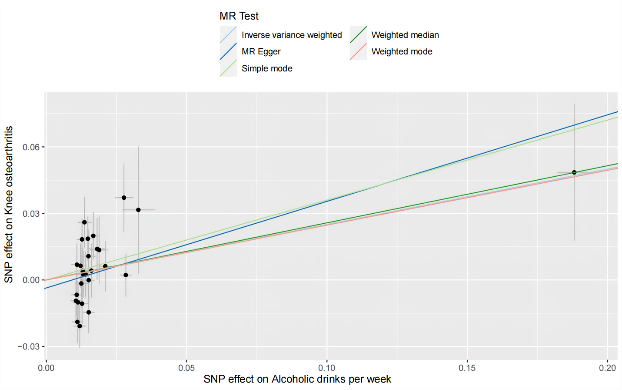

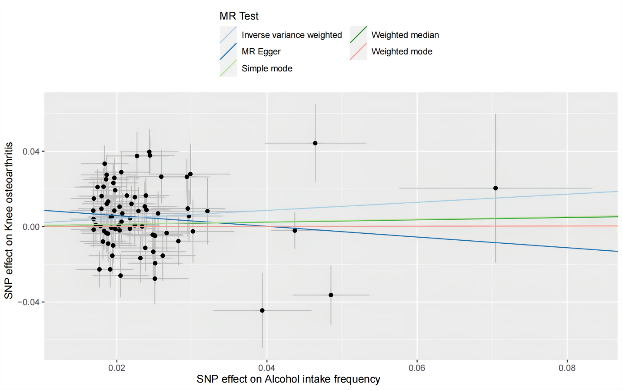

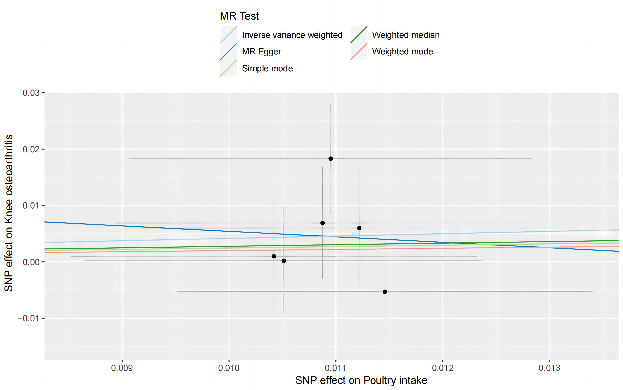

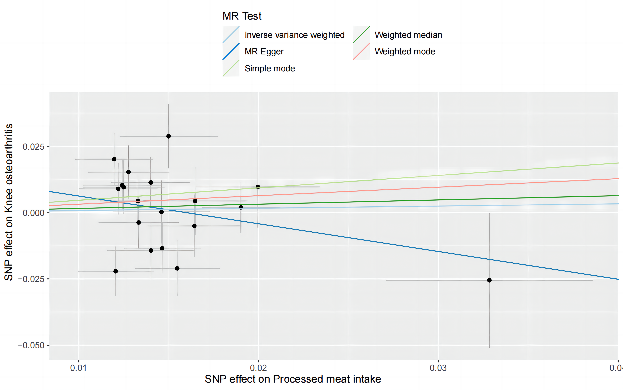

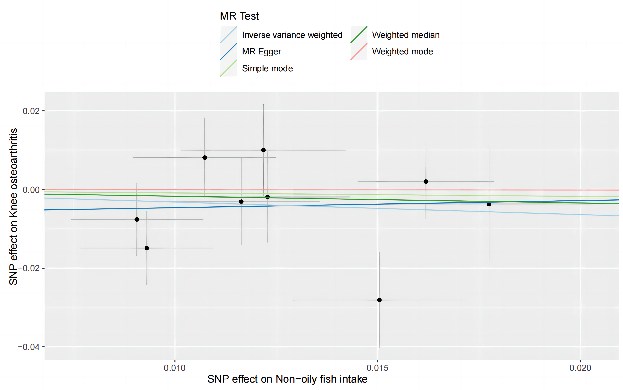

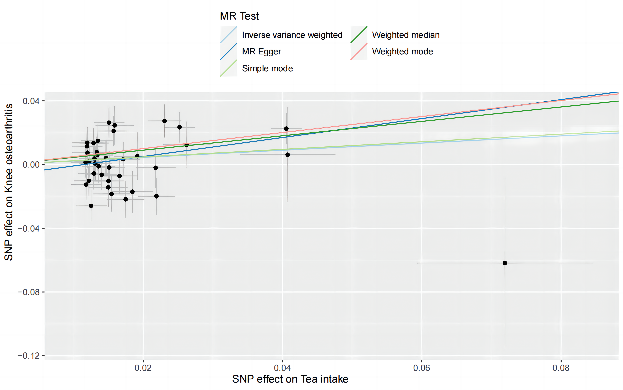


S1 Fig: Scatter plot of the causal relationship between diet and KOA. Scatter plot of negative results.

S2 Fig:


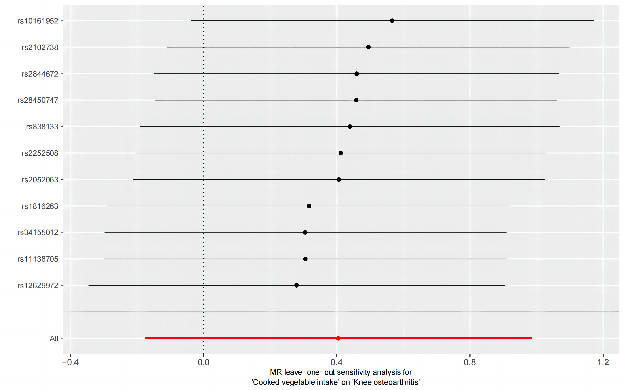

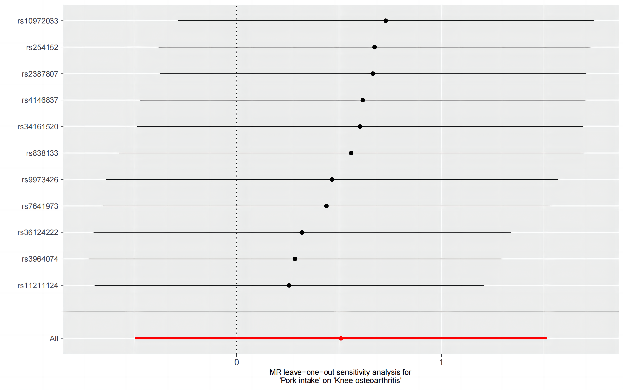

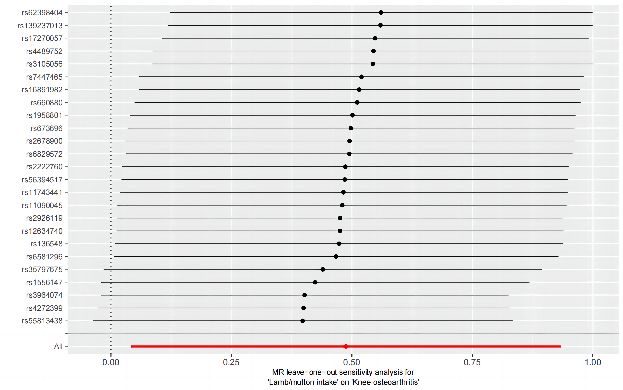

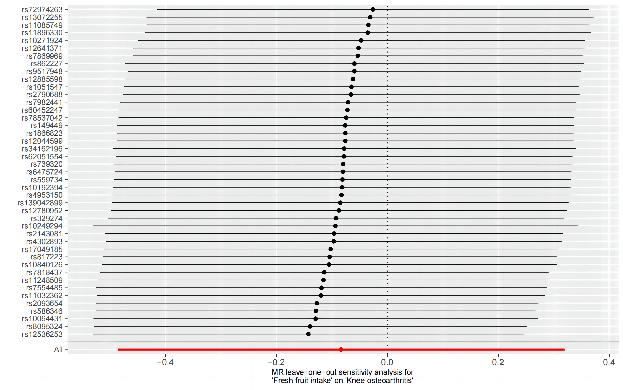

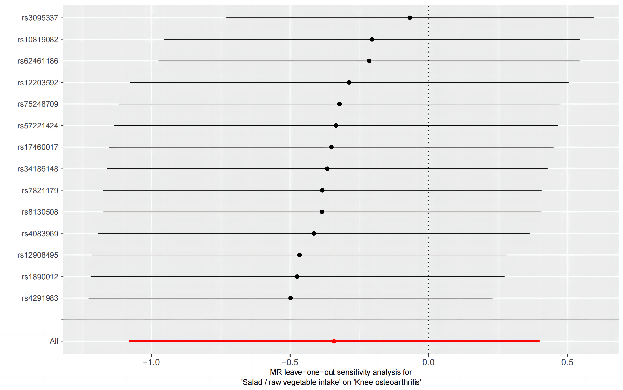

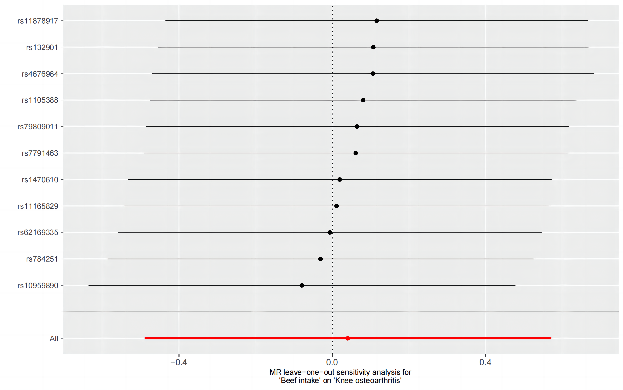

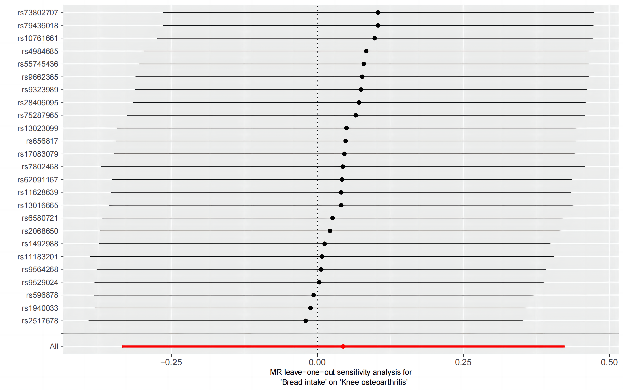

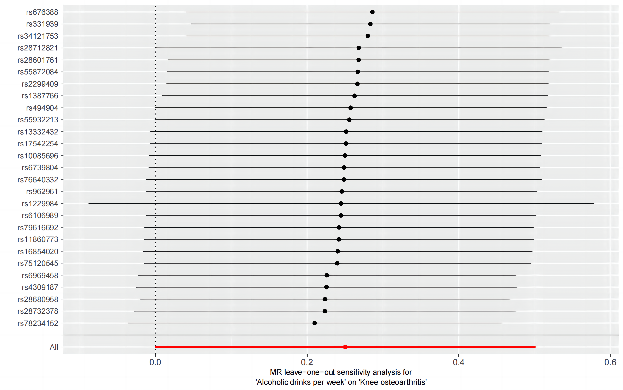

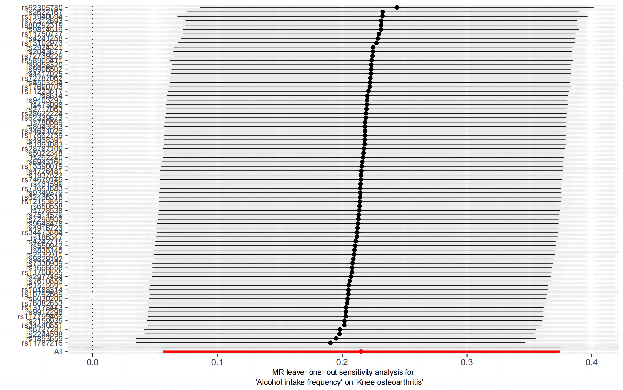

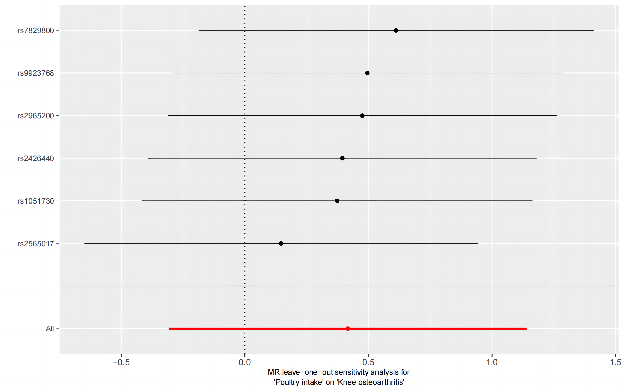

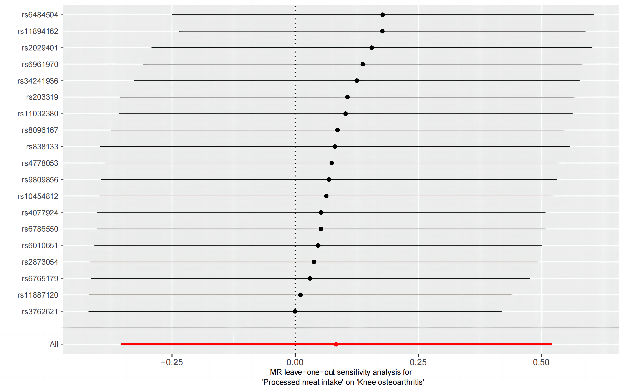

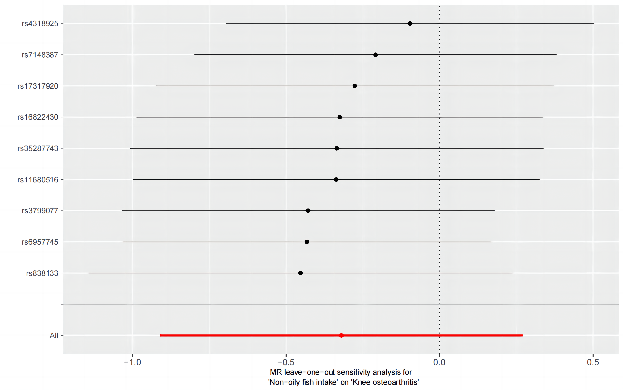

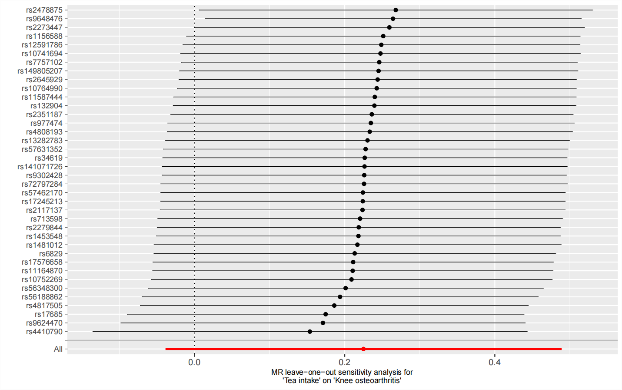


S2 Fig: Leave-one-out analysis of the negative results of diet and KOA. Red lines represent estimates from IVW tests. IVW: Inverse Variance Weighted.
